# An Internet-Based Intervention (Mamma Mia) for Postpartum Depression: Mapping the Development from Theory to Practice

**DOI:** 10.2196/resprot.4858

**Published:** 2015-10-12

**Authors:** Filip Drozd, Silje Marie Haga, Håvar Brendryen, Kari Slinning

**Affiliations:** ^1^ National Network for Infant Mental Health Centre for Child and Adolescent Mental Health, Eastern and Southern Norway Oslo Norway; ^2^ Norwegian Centre for Addiction Research University of Oslo Oslo Norway; ^3^ Department of Psychology University of Oslo Oslo Norway

**Keywords:** early intervention, Internet, intervention mapping, Mamma Mia, postpartum depression, pregnancy, well-being

## Abstract

**Background:**

As much as 10-15% of new mothers experience depression postpartum. An Internet-based intervention (Mamma Mia) was developed with the primary aims of preventing depressive symptoms and enhancing subjective well-being among pregnant and postpartum women. A secondary aim of Mamma Mia was to ease the transition of becoming a mother by providing knowledge, techniques, and support during pregnancy and after birth.

**Objective:**

The aim of the paper is to provide a systematic and comprehensive description of the intervention rationale and the development of Mamma Mia.

**Methods:**

For this purpose, we used the intervention mapping (IM) protocol as descriptive tool, which consists of the following 6 steps: (1) a needs assessment, (2) definition of change objectives, (3) selection of theoretical methods and practical strategies, (4) development of program components, (5) planning adoption and implementation, and (6) planning evaluation.

**Results:**

Mamma Mia is a fully automated Internet intervention available for computers, tablets, and smartphones, intended for individual use by the mother. It starts in gestational week 18-24 and lasts up to when the baby becomes 6 months old. This intervention applies a tunneled design to guide the woman through the program in a step-by-step fashion in accordance with the psychological preparations of becoming a mother. The intervention is delivered by email and interactive websites, combining text, pictures, prerecorded audio files, and user input. It targets risk and protective factors for postpartum depression such as prepartum and postpartum attachment, couple satisfaction, social support, and subjective well-being, as identified in the needs assessment. The plan is to implement Mamma Mia directly to users and as part of ordinary services at well-baby clinics, and to evaluate the effectiveness of Mamma Mia in a randomized controlled trial and assess users’ experiences with the program.

**Conclusions:**

The IM of Mamma Mia has made clear the rationale for the intervention, and linked theories and empirical evidence to the contents and materials of the program. This meets the recent calls for intervention descriptions and may inform future studies, development of interventions, and systematic reviews.

## Introduction

### Background

The postpartum period represents a vulnerable time where the woman is at increased risk of mental disorders [1]. Between 10% and 15% of women experience moderate to severe depressive symptoms during pregnancy and after childbirth [2-5]. Depressive symptoms postpartum can have severe consequences and lead to negative parenting behaviors [6], child psychopathology in general [7], and increase the risk of depression among partners [8]. The prevention and treatment of postpartum depression (PPD) is thus essential for a mother, her infant, and the family’s mental health and well-being.

Psychological treatments for PPD are effective [9]; however, many women living with PPD are not identified and do not receive adequate treatment (see eg, [10]). This is a serious concern not only for the affected families, but also for the society as a whole. The social costs of mental illnesses for each annual cohort of births are estimated to be approximately £1.2 billion [11]. Consequently, there is a need to reach pregnant women and provide accessible evidence-based help and support to prevent PPD.

Internet interventions may be feasible in preventing and treating PPD with its potential for high reach. In fact, many pregnant women use the Internet to search for pregnancy-related information such as fetal development or childbirth [12]. Trend data show that 66-75% of Norwegian women in childbearing age (ie, 16-44 years) searched for health information online in the past 3 months in 2013 [13], and in the United States, 19% of Internet users report to have searched for information about pregnancy and childbirth [14]. Many women with PPD also express an interest in Internet interventions and report that they would use the Internet to learn coping strategies for PPD [15].

Recent studies have demonstrated the acceptability and feasibility of Internet interventions for PPD [16-20]. Results from the first randomized trials also offer promise for Internet interventions as an effective treatment for PPD [21-23]. However, as in almost all intervention research, intervention descriptions tend to be rather brief and general, and often confined to a few paragraphs in the methods section. This makes it difficult for researchers to identify active ingredients and practically impossible for intervention designers to make informed decisions about future intervention development and how to improve existing interventions. This is a serious concern for intervention research because it violates one of the basic premises of research—*replication* of studies. In other words, the reporting of interventions and conduct of intervention studies generally fail to contribute toward a cumulative science of Internet interventions.

### Objective

The aim of this paper was therefore to provide a systematic and comprehensive review of an Internet intervention for the prevention of postpartum depressive symptoms and enhancement of subjective well-being. While previous research has mainly focused on the treatment of PPD, this intervention has a strong preventive focus considering the potential for social savings (see the earlier discussion). We used the intervention mapping (IM) protocol, which outlines the path from recognition of a need or problem to the identification of a solution [24]. The end product constitutes a comprehensive blueprint of the intervention and a detailed treatment rationale that may facilitate replication, support the interpretation of subsequent implementation and evaluation studies, and ease the comparison of the treatment rationale across interventions [25,26].

## Methods

### Intervention Mapping

IM is a tool that systematizes and integrates theory, empirical evidence, and information collected from the target population when designing health promotion programs. It makes the development of interventions transparent and provides an explicit report of all the decisions and considerations throughout the intervention process. There are 6 fundamental steps in the IM process: (1) the conduction of a needs assessment or problem analysis; (2) the definition of proximal program objectives based on scientific analyses of a given health problem and its predictors; (3) the selection of theory-based intervention methods and practical strategies to change (determinants of) health-related behaviors; (4) the production of program components, design, and production; (5) the anticipation of program adoption, implementation, and sustainability; and (6) the anticipation of process and effect evaluation. Each step comprises several tasks and the completion of 1 task guides the completion of a subsequent task. Although IM is presented as a series of steps, Bartholomew and colleagues [24] look at the planning process as iterative rather than linear, meaning that program planners move back and forth between the various tasks and steps. The process is also cumulative in the sense that each step is based on previous steps, and the failure to attend to important aspects in any given step may lead to mistakes and inadequate decisions in subsequent steps.

### Post Hoc Application of the IM Protocol

The IM framework greatly influenced the development of Mamma Mia, which makes a post hoc analysis both feasible and informative. Ideally, the IM protocol is used a priori in intervention development. In this study, however, the IM procedure was applied in a post hoc manner. Previous studies illustrate, though, that a retrospective IM-based analysis can also be a useful tool for post hoc description of interventions [27,28]. Specifically, it may point toward weaknesses in the intervention development process and the intervention itself, thereby anticipating any potential threats and issues that may arise during the implementation and evaluation. Nonetheless, an application of the IM protocol after the development of the intervention has taken place means that the actual course of actions deviate to a certain extent from what is prescribed by the IM protocol. Most notably, this concerns Steps 5 and 6 in the IM protocol where program adopters and implementers were not included in the intervention development in the strictest sense of the IM protocol; additionally, the evaluation of the intervention is mostly focused on the effectiveness of the intervention, rather than process evaluations of the development. Any deviations from the IM protocol are noted throughout in the results.

## Results

### Step 1: Needs Assessment

A thorough exploration of the health problem, referred to as the needs assessment, is an inherent part of the IM framework. The result of a needs assessment illustrates how prevalent the problem is and what factors are associated with it. In this study, the health problem is PPD, and the challenge is that many women who experience depressive symptoms receive no counseling or support. An exploration of the literature suggests that many women report to be unfamiliar with symptoms of PPD and do not realize that they may be suffering from depression [29]. Symptoms of PPD may be difficult to distinguish from symptoms normally observed in postpartum women such as tiredness, changes in sleep, appetite, and sexual desire (ie, symptoms that are normally observed in women after giving childbirth and taking care of a newborn baby), making it also difficult for health professionals to detect women with PPD. This may explain, in part, why women often fail to seek help for their PPD (ie, 17-25%) [30]. Other barriers to help seeking include women’s inability to disclose their feelings, for example, because of shame or fear of losing custody, and health professionals’ reluctance to respond to the mothers’ emotional and practical needs [31]. In addition, consultations with general physicians (GPs), midwives, and public health nurses (PHNs) at well-baby clinics tend to be rather brief in the prenatal and postnatal periods (ie, regular appointments in Norway are scheduled to last about 15-20 minutes), thereby making it difficult to detect and respond to PPD. A preventive intervention, however, can not only prevent the development of depressive symptoms, but also help a woman become aware of and identify symptoms of depression, and possibly encourage her to seek help and support.

As in any preventive intervention, it is important to target risk and protective factors that may influence the onset and development of PPD. However, most studies have typically emphasized and identified risk factors that are hard or even impossible to modify such as a previous history of depression, negative life events, and certain demographic characteristics [32]. Thus, as part of the current needs assessment, we conducted 2 studies to investigate the contribution of modifiable psychological risk factors associated with perinatal depressive symptoms and well-being further. In a longitudinal study, self-efficacy, certain cognitive emotion regulation strategies (eg, rumination, self-blame, and positive reinterpretation), perceived available support, and need for support were found to predict the rate of postpartum depressive symptoms [33]. Interviews with new mothers largely confirmed these findings, but also highlighted that the woman’s expectations and approach to motherhood influenced her feelings of depressed mood and well-being. Specific expectations and a high need for mastery and planning (ie, controlling) made women more vulnerable and at-risk for experiencing lower mood and subjective well-being, compared with women who were more relaxed [34]. From an intervention perspective, it thus became important to target these modifiable psychological risk factors.

A recent and large Norwegian population-based study showed that relationship satisfaction protects against emotional distress during pregnancy [35], whereas dissatisfaction with the partner relationship predicted maternal emotional distress [36]. A satisfying relationship is important to prevent depression and to retain and increase life satisfaction [37], especially because both relationship and life satisfaction tend to decrease after childbirth and remain below prebirth level for several years [38]. Although Norwegian women may be more satisfied with their lives during pregnancy and following birth in general than women in other countries, it is still common that life satisfaction drops after the “baby honeymoon” period [39]. Hence, improving relationship satisfaction and well-being are major targets for preventing PPD in both pregnant and postpartum women.

Finally, parental insensitivity, which refers to disengagement, intrusiveness, or noncontingent responding, to the infant’s cues is associated with PPD [40,41]. Increasing parental sensitivity thus makes up a final key modifiable psychological factor that should be targeted in an intervention. This is important because lack of parental sensitivity and insecure attachment relationships can instigate cycles of transactional or bidirectional effects that can both exacerbate parental symptoms of depression and increase the risk of internalizing and externalizing problems in infants [42-44], difficulties that may continue into late adolescence [45,46]. Prenatal depression and PPD may affect parenting capabilities such as parental sensitivity, which may in turn instigate the development of an insecure mother-child attachment relationship [47,48]. Thus, promoting healthy and supportive attachment relationships between parents and their infants, thereby increasing parental sensitivity, may be of great importance for parents and infants’ long-term adjustment and mental health.

### Step 2: The Performance and Change Objectives of Mamma Mia

The second step in IM is about defining the overall goals of an intervention and the performance and change objectives, which, in turn, specify how the overall goals can be achieved. To arrive at these objectives, the IM protocol suggests a procedure in which the overall goals are broken down into subgoals (ie, performance objectives) and correlates of subgoals are identified (ie, determinants). Change objectives are then constructed to target the determinants of the performance objectives. In short, change objectives are essentially what the user has to change or learn to attain the performance objectives.

#### The Overall Goals of the Intervention

The intervention (Mamma Mia) was designed as a universal preventive measure that could be offered to all pregnant women with the primary goals to (1) prevent the onset or development of depression and (2) enhance subjective well-being during the prenatal and postnatal period (ie, starts in gestational week 18-24 and lasts up to 6 months after giving birth). A secondary goal was to ease the transition of becoming a mother by providing knowledge, techniques, and support during pregnancy and after birth. The reason for starting in the second trimester is that expectant mothers can then be reached as early as possible, they have a reduced risk of spontaneous miscarriage, all women attend ultrasound at this time (ie, see their baby) and start forming prenatal attachment to their baby, and because the prevalence of prenatal depression is as common as postpartum. Six months after birth is the end point of the intervention as postpartum depressive symptoms tend to fade around this time [1]. However, to prevent postpartum depressive symptoms and increase well-being are not complete descriptions of the desired outcome. The specific behaviors required to accomplish the desired outcomes need to be described in greater detail. To do so, we need to consider some basic facts about the psychological process of expecting a child and taking care of a newborn baby.

#### The Performance Objectives

The performance objectives of the Mamma Mia intervention are presented in the left column in Table 1. These are specifications of the overall goals and defines more clearly what it entails to prevent PPD in behavioral terms at the personal and interpersonal levels. It is, for instance, important that the mother regularly screens herself for depressive symptoms and is encouraged to seek help and support, and provided with immediate and additional on-screen support (ie, “just-in-time” therapy; see PO4 in Table 1). In addition, due to the already comprehensive approach to PPD in Mamma Mia, and the increasing complexity and additional costs associated with differentiation of subgroups, it was not feasible to differentiate the population at this stage in the development.

Overall, the needs assessment identified that for women to be able to prevent and alleviate perinatal depressive symptoms, they have to successfully manage the transition to parenthood. This also entails engaging in relationship- and health-promoting social and mental activities, both at a personal level and in relation to the woman’s baby and her partner. The woman regularly needs to assess how she is doing and, if necessary, request help and support. For women who develop depressive symptoms, it is important that she is provided with immediate help and support to take the edge off the symptoms, as soon as possible, to prevent any adverse consequences as well as the onset of a more serious clinical depressive disorder or recurrent depressive episodes.

#### Personal and Interpersonal Determinants

Once the performance objectives were specified, we returned to the needs assessment to identify modifiable factors that in some way cause or can prevent perinatal depressive symptoms (see top row in Table 1). At the personal level, knowledge about or awareness of perinatal depressive symptoms is probably the first prerequisite for preventing depressive symptoms (see eg, PO4 in Table 1). Becoming aware of perinatal depression, the woman may need to adjust or relax certain expectations and attitudes that otherwise may nourish symptoms of depression (see eg, PO1 in Table 1), and become more tolerant and self-accepting of her pregnancy and becoming a parent. At the interpersonal level, it is important that the woman gets to know her baby and forms an emotional bond during pregnancy, as this predicts secure attachment 1 year postpartum [49] (see eg, PO2 in Table 1). This may help her establish an early relationship with her baby characterized by amazement and enjoyment rather than disruptive behaviors or a lack of contact between the mother and her child. Furthermore, by taking care of her partner relationship, this may act as a buffer against distress during pregnancy and the postpartum period, and be the first step in reaching out for help in cases where a woman may feel burdened or saddened (see eg, PO3 and PO5 in Table 1).

#### The Change Objectives

The next step was to develop and specify the change or learning objectives. The change objectives constitute the actions the mother has to do to carry out the performance objectives, and are a response to the question “What do intervention users need to change or learn to accomplish the performance objectives?” The performance objectives, determinants, and change objectives for the Mamma Mia intervention are summarized in Table 1. The cells in Table 1 thus constitute the building blocks or change processes in the intervention. This can be seen as an overview of the active ingredients of the intervention and as a blueprint of the theoretical treatment rationale.

In reviewing the literature and analyzing the problem of PPD, it became apparent that there is a great need for psychoeducational information (ie, knowledge) about postpartum depressive symptoms and the importance of the couple’s relationship and emotional bonding between a mother and her child during pregnancy and the postpartum period. Many women report being unfamiliar with symptoms of depression during pregnancy and after childbirth, and express or hold certain expectations or beliefs that can be counterproductive with regard to one’s mental health. For example, learning that a depressed mood usually makes people more socially withdrawn and quickly lose interest in social activities can result in a lack of interactivity and emotional bonding with the baby. This may cause some women to attribute failures to connect with the baby to personal and stable characteristics by themselves rather than realizing that most babies may need up to 10 seconds to respond to parent-initiated interaction. Thus, by learning simple attachment strategies such as “being with baby” and the principle of “wait, watch, and wonder,” the woman may prevent the spiraling of such vicious transactional cycles.

In the wider context, this matrix of change objectives functioned as a backbone for the selection of theories and methods in the translation of these into the actual intervention.

**Table 1:** Performance and change objectives for the Mamma Mia intervention.

	Determinants			
Performance objectives	Knowledge	Expectancies and attitudes	Attachment, emotion regulation, and help seeking	Relationship satisfaction and communication skills
PO1: Cope adaptively with becoming a parent	K1.1: Understand that mixed feelings are normal postpartum	EA1.1: Accept that experiencing the maternity blues is normal	AEH1.1: Prepare friends and family for the expecting baby and upcoming life changes	RC1.1: Demonstrate the skill to effectively communicate and share needs and expectations toward partner
	K1.2: Acknowledge that detailed planning can be counterproductive	EA1.2: Let go of the need for rigorous and detailed planning and control		
	K1.3: Recognize that the postpartum period is hectic, and that it is important to be realistic about what one can achieve	EA1.3: Believe that breast-feeding is a skill that needs to be learned, and that there are alternative options		
	K1.4: Learn about alternatives to breast-feeding			
PO2: Engage in positive parent-infant interactions	K2.1: Understand how PND can interfere with bonding between a mother and her infant	EA2.1: Reflect confidence in parenting ability	AEH2.1: Experience “being with baby”	
	K2.2: Learn about infant development	EA2.2: Attribute failures to connect with infant to situational factors	AEH2.2: Identify and recognize the sleep-wake cycles of infants	
	K2.3: Become aware of the infant’s attention and communication skills	EA2.3: State that infants need time to react and respond	AEH.2.3: Demonstrate parent-child interaction and engage in appropriate attachment behaviors	
		EA2.4: Set realistic personal standards and expectations for the prepartum and postpartum period	AEH2.4: Utilize the principle of “wait, watch, and wonder” in interactions with her baby	
		EA2.5: Accept “good enough” parenting		
PO3: Engage in proactive and positive physical and mental activities	K3.1: Know the rationale for the positive psychological approach and learn the benefits of engaging in positive activities	EA3.1: Feel positive about involving the partner in preparations and taking charge	AEH3.1: Use techniques to enhance subjective well-being	RC3.1: Correctly perform exercises that can increase relationship satisfaction
	K3.2: Understand the pros of enhancing the partner relationship during pregnancy		AEH3.2: Practice relaxation and being present minded	RC3.2: Demonstrate more positive emotions toward partner while decreasing the expression of negative emotions
	K3.3: Understand that certain beliefs or assumptions about partner relationship are false or myths		AEH3.3: Make a list, plan, and engage in pleasant activities	RC3.3: Demonstrate a set of principles and use techniques for improving partner communication
			AEH3.4. Engage in physical activity	
PO4: Get help and support if depression is indicated	K4.1: Know that there are effective methods for managing depressive symptoms	EA4.1: Feel positive about and see the need to screen for depressive symptoms	AEH4.1: Ask for partner support	
	K4.2: Describe potential symptoms of postnatal depression		AEH4.2: Call mental health hotline	
	K4.3: Realize that social withdrawal from partner and others is a part of the problem		AEH4.3: Contact general physician	
			AEH4.4: Active and continued participation in Mamma Mia	
PO5: Cope adaptively with symptoms of depression	K5.1: Learn to identify certain maladaptive ways of thinking and behaving	EA5.1: Expect that using the techniques learned in Mamma Mia can be beneficial	AEH5.1: Learn a set of techniques to improve mood	See RC1.1 and RC3.1-3.3
		EA5.1: Feel positive about asking for help and support, and expect that it can be beneficial	AEH5.2: Change or replace ineffective mood strategies	
			See also change objectives for PO2 and change objectives AEH4.1-4.4	

### Step 3: Theory-Based Methods and Practical Strategies

In the third step, the IM protocol addresses the selection of theoretical methods based on the change objectives identified in Step 2. Selected methods are then translated into practical strategies giving consideration into “what should be done” and “how it should be done” (ie, strategies for use). As seen in Table 2, both self-regulation and information processing theories served as the foundation for the development of practical strategies to influence knowledge, expectancies, and attitudes. These represent a conscious effort to become aware of and reflect on the physiological and psychological processes during pregnancy and after childbirth, and encourage a more flexible, open-minded attitude toward a range of beliefs and ways of thinking. Although knowledge or expectancies may not translate directly into behavior change, they provide a framework for understanding the importance of engaging in (self-)caring behaviors during the perinatal period, which may translate into behavior change (eg, finding alternatives to breast-feeding or selecting positive persons for support). In the remaining part, methods and strategies for use are presented and reorganized in accordance with the overall goals and determinants in the intervention from the user’s perspective.

**Table 2:** Mamma Mia determinants, methods, and strategies for use.

Determinants	Methods	Strategies for use
Knowledge	Consciousness raising (TTM^a^)	Psychoeducation, guidelines, and recommendations are often followed by, for example, reflective questions intended to raise awareness about certain counterproductive expectancies or attitudes (eg, “I can’t ask anybody for help, I should be able to take care of my own baby”)
	Active learning (ELM^b^ and SCT^c^)	Psychoeducational information, cognitive and behavioral assignments, brief and many learning moments with repeated content over time to provide opportunities for rehearsal.
	Elaboration (ELM^b^)	Information should be relevant, easily understandable, and rewarding to follow. Aligned with the chronology of the physiological and psychological processes during pregnancy and postpartum.
Expectancies and attitudes	Goals/personal standards (SRT^d^)	Promote acceptance of a lesser need for detailed planning and rigorous control (eg, unexpected events may occur during birth).
	Normalization (NSI^e^)	Normalize mixed feelings, potential failure to breast-feed, and feeling low on energy, which can assist with relaxation.
	Self- and environmental re-evaluation (TTM^a^)	Stimulate appraisal to self-assess depressive symptoms, reinforce partner involvement, reinforce early parent-child bonding (eg, fantasy baby).
	Verbal persuasion (SCT^c^)	Communicate optimism about users’ parenting abilities, benefit of participating in Mamma Mia, and support-seeking behavior.
Attachment, emotion regulation, and help-seeking	Newborn Behavioral Observation	Video demonstrations of infant sleep-wake cycles and social interactive skills, homework assignments.
	Circle of security	Illustrated graphics to help parents understand their baby’s needs and activate appropriate attachment behaviors.
	Positive psychotherapy (PPT^f^)	Provide concrete tasks that focus users’ attention on all the good things in life to enhance positive emotions, engagement, and a sense of meaning (eg, gratitude exercises); homework assignments.
	Mindfulness (PPT^f^)	Audio-taped instructions that foster being in the moment, which are provided as downloadable audio files for personal use; homework assignments.
	Behavioral activation (PPT^f^)	Recommendations for physical activity during pregnancy and after childbirth. Compile a list of pleasant activities and schedule pleasant activities over the course of the intervention.
	Stress and coping social support theory/relational regulation	Edinburgh Postnatal Depression Scale to assess depressive symptoms, encourage asking for partner support and/or general physician, provide a phone number to a mental health hotline.
	Metacognitive therapy	Audio-guided instructions and exercises (eg, attention training technique) to induce a state of awareness of internal events (eg, excessive worry) without responding cognitively, emotionally, or behaviorally; homework assignments.
Relationship satisfaction and communication skills	Gottman’s method (couples therapy)	Couple exercises and homework to build closeness with partner, create a supportive relationship, and learn to manage conflicts (eg, softening technique); homework assignments.
	Prevention and relationship enhancement program	Video demonstrations of communication and problem-solving skills (eg, speaker-listener technique); homework assignments.
	Nonviolent communication	Practice distinguishing observations from interpretation of actions, identifying and expressing one’s feelings and needs in a nondemanding way, and be given performance feedback.

^a^TTM = transtheoretical model

^b^ELM = elaboration likelihood model

^c^SCT = social cognitive theory

^d^SRT = self-regulation theory

^e^NSI = normative social influence

^f^PPT = positive psychotherapy

#### Depressive Symptoms

Methods and strategies addressing depressive symptoms mainly pertain to help seeking in Table 2 where the Edinburgh Postnatal Depression Scale (EPDS) was used, so that mothers can self-assess their depressive symptoms in the present intervention. The EPDS has been validated for Internet administration [50,51] and a systematic review shows that it reduces the probability for postpartum depressive symptoms [52], especially in combination with supportive counseling [53]. Therefore, women who screen positive (ie, EPDS score ≥ 10) are provided with immediate help and support based on metacognitive therapy (MCT) [54]. In MCT, a client deals with the cognitive mechanisms (eg, worry or rumination) that lead to emotional problems, rather than the content of specific negative thoughts, feelings, or beliefs, as in cognitive therapy. MCT can be effective for PPD [55], and appears promising for delivery in a digital format considering that some techniques and exercises are administered as audio-taped files, even in face-to-face sessions. For women who screen negative, evidence suggests that *universal* interventions may have a preventive effect in postpartum women [56,57]. Thus, it is equally important to be concerned with well-being and the maintenance of healthy mental practices or habits, even among nondepressed women.

#### Maternal Subjective Well-Being

Methods for emotion regulation were approached from a positive psychological perspective (see Table 2). In general, a positive psychological approach is concerned with doing more of what is right or healthy (ie, prescriptions about the good life), rather than correcting what is wrong (ie, symptom substitution). These methods have been previously documented to have a positive effect on well-being among healthy, normal adults, as delivered by Internet [58]. It has been suggested that disseminating a broad range of positive psychology interventions (PPIs) online, tends to increase their use and effectiveness [59,60]. Multiple PPIs were thus drip fed throughout the current intervention, although the intervention conveys that users should not use all the PPIs constantly or simultaneously, but rather try each PPI and use those that they find personally most relevant and useful. Consequently, a diverse range of PPIs were administered in the present intervention such as mindfulness (eg, mindful breast-feeding) [61], gratitude (eg, 3 good things) [62], acts of kindness [63], and other exercises (for examples, see [64]). It should be noted that some of the included PPIs were designed for mastery of adverse situations (ie, expressive writing and cognitive restructuring; see eg, [65]) and that several of these exercises were also adapted to support the processes involved in strengthening the couple relationship (eg, giving your partner a compliment or savoring past, positive relationship experiences).

#### Partner Relationship

As presented in Table 2, the intervention draws upon principles from Gottman’s method for healthy relationships [66,67] and the Prevention and Relationship Enhancement program [68] to strengthen the couple relationship. Both methods are concerned with the basic ways in which couples communicate and manage conflicts or problems (eg, avoiding criticism and listening actively). The present intervention thus includes methods for effective communication such as sharing expectations with one another, showing an interest in one’s partner (eg, building love maps), expressing positive emotions (eg, “I know you love me when...”), reflecting about how one argues and how these may be perceived by the partner, softening start-ups in conflicts, and discussing problems (eg, speaker-listener technique). Finally, to support conflict management and help couples resolve problems, the 4 principles of nonviolent communication were taught to help users (1) distinguish the assessment of or feelings evoked by the action of others, (2) identify and express their feelings and (3) needs, and (4) convey requests in a noncoercive or demanding way [69].

#### Parental Sensitivities and Mother-Infant Relationship

To sensitize mothers to their infants’ competencies and to promote the development of a healthy mother-infant relationship, the intervention includes several items and concepts from the Newborn Behavioral Observation (NBO) system [70] and circle of security (CoS) [71]. The NBO system is an infant-focused, relationship-based method that includes, in this intervention, demonstrating infants’ sleep-wake cycles, and their abilities to respond to stress, self-regulate, and socialize. Concepts from the CoS intervention were used in a complementary manner to NBO, with a focus on parents and their ability to reflect on their own and their infant’s behaviors, thoughts, and feelings, and respond appropriately to the infant’s signals. In the current intervention, it includes demonstrating the concepts of a secure base and safe haven, which represent the baby’s need for exploration and learning and its need for protection and comfort, respectively. The principle of “watch, wait, and wonder” is used to support parents in their work with the concepts in CoS [72], and it should be noted that the current intervention also focuses on attachment processes during pregnancy. Taken together, the current intervention focuses mainly on the social and interactive components of NBO and CoS where the goal is to support mothers learn to understand their infant and respond in a positive and developmentally supportive manner (Table 2).

### Step 4: Program Components and Materials

#### Information and Communication Technology

Today, information and communication technology represents a promising channel for dissemination of Internet interventions in Norway. Most Norwegians have access to, and use, the Internet. On an average day, 83% of women in the population report having used the Internet; whereas 96% and 100% of women in childbearing age (ie, 16-44 years) reported having used email and the Internet in the last 3 months in 2014, respectively [13]. Given that many women in childbearing age use the Internet and are so-called eHealth information seekers as described earlier, the decision to develop an Internet intervention was taken at the very beginning of the process. One important aspect to this decision, however, is that 73% reported in 2013 to have access to mobile phones, and 61% of the population had access to tablets [73]. Thus, it was considered important to make the intervention platform independent (ie, independent of any specific hardware or software) to ease the accessibility and use of the program (ie, available on smartphones and tablets). The Internet was considered an appropriate and cost-effective medium for preventing PPD, especially given that the long-term goal is to offer the intervention to all pregnant women in Norway (ie, about 60,000 women/year) [74]. Furthermore, it was to be a fully automated self-help intervention, which is consistent with the aim of developing a primary preventive intervention for perinatal depressive symptoms.

#### Tunneled Design and Program Structure

The 2 core organizing principles for the delivery of intervention content are the tunneled information architecture and noise reduction (see Multimedia Appendix 1) [75]. The first principle, the tunneled design, entails that information is presented in a predetermined sequence page-by-page and session-by-session. It can have a negative effect on users’ perceptions of efficiency, but seems to have a positive effect on intervention adherence and acquisition of knowledge [76]. The second principle, noise reduction, means that information only becomes available at the right time during pregnancy and postpartum rather than being available all the time. For example, in its simplest form, information about fetal development is delivered according to gestational week. This is done to avoid distractions and to minimize cognitive load on users [77]. Consequently, user navigation is, most often, limited to only “back” and “next” options. Several Internet interventions have used a tunneled design and reduction, demonstrating the feasibility and promise of such an information architecture for mental health and behavior change [78-81].

The intervention is used individually by the mother with health professionals, partners, or other actors only involved indirectly through the mother, but with no direct access to the program. For access to each session, users receive an email with a unique link. By clicking on the link, users are directed to a sequence of websites that are unique for that particular session. In the prenatal phase, emails with sessions are scheduled on a weekly basis, while sessions are scheduled 3 times a week in the active postpartum phase (ie, weeks 3-9). This is followed by a low-intensity phase with weekly and eventually monthly sessions. The intervention starts in gestational weeks 18-24 and lasts until the baby is about 6 months. In total, the intervention consists of 44 sessions over a period of 11.5 months. Each session is designed to take about 10 minutes and must be completed before users can access the next session. This is done to ensure that relevant information has been reviewed and to create continuity and a narrative in the program. If a mother discontinues a session, she is required to complete the previous session before she can move on to the current session. If the mother is on schedule, the session will end by directing her to the intervention home page. On average, a typical website during a session consists of 80-100 words and a typical user may have 10-15 pages per session (ie, the exact number of pages per session may vary due to tailoring). For a demonstration, see [82].

A session may utilize various functionality and interactivity to engage users, but typically consists of psychoeducational information, interactive tasks, and cognitive or behavioral homework assignments (for examples, see Multimedia Appendix 2). Psychoeducation is most often text-based information supplemented with printable documents, graphical illustrations, and video demonstrations. As an example, mothers are provided with descriptions of the infant’s behavioral states followed by video demonstrations (see “Newborn Behavioral Observation” under “Methods” in Table 2). Interactive tasks typically include on-screen activities such as audio-guided instructions, quizzes, questionnaires, and written and reflective assignments. Examples of interactive tasks are filling in the EPDS questions and performing audio-guided mindfulness exercises (see “Self-and Environmental Re-evaluation” and “Mindfulness” in Table 2). Mindfulness involves experiential on-screen and homework exercises presented repeatedly. Mindfulness and re-evaluation represent a good example of how users are actively engaged in their change process where information is often elaborated and rehearsed over time (see “Active Learning” and “Elaboration” in Table 2). Homework assignments often accompany interactive tasks and are always related to the topic of a particular session. They may consist of reflecting upon why the woman reported to be feeling down lately and whether she needs someone to talk to, and practicing mindfulness in everyday life. A final example demonstrates how methods for the partner relationship are translated into the actual intervention; a mother is provided with psychoeducational text-based information about how conflicts may escalate and is allowed to watch a video demonstration of a couple going into an argument about trivial issues (eg, leaving dirty laundry on the bathroom floor). For homework, she is given a downloadable and printable document where she and her partner are supposed to discuss how they usually argue and how these conflicts are perceived. The lesson learned is that all couples argue from time to time but it is not the number of conflicts that determines whether the couple’s relationship will be in a satisfying relationship or not, but rather how these conflicts are handled and perceived. Two sessions later, she is provided with a blueprint for the softening technique to learn to handle conflicts in a gentler and more appropriate manner—see “Gottman’s Method (Couples Therapy)” and “Prevention and Relationship Enhancement Program” in Table 2.

At the end of each session, the user is directed to the intervention home page (see Multimedia Appendix 1). This home page contains an overview of the entire intervention where users may review completed sessions and retrieve all learning materials. The home page employs a top-down hierarchical information architecture and helps users find desired content by identifying the broad topics for each sessions. It is interesting that the tunneled design, on the one hand, restricts a user’s navigational freedom, but may foster an interactive dialog that would otherwise be difficult to mimic or require more costly and early stage technology (eg, relational agents, semantic Web or natural language processing). On the other hand, the hierarchical design relaxes the restraints imposed by the tunneled design and allows users to freely explore and rehearse completed contents.

#### Personification and Tone-of-Voice

In its most rudimentary form, the Internet is a text-based medium. However, even static text material can mimic features of human-to-human dialog that can foster a sense of relationship or alliance between an intervention and the user [83-85]. This may, in turn, be positively related to the use and impact of the intervention [86,87]. A review by Kennedy and colleagues [88] found that users do have a preference for empathy and other relational behaviors in dialog systems for various health topics. Therefore, the intervention is embodied or personified by depicting 3 different persons alongside text and other interactive components, with each representing their special topic. The 3 persons are presented in 3 separate “rooms,” which organize and structure the content and intervention materials: (1) the mother’s room, (2) the couple’s room, and the (3) baby’s room. The agent in the mother’s room conveys information about the mother by screening for depressive symptoms and administering the well-being component, as described earlier. The 2 remaining agents provide information and exercises related to the couple’s relationship and parental sensitivity, respectively.

The information is conveyed in a pleasant, but informal tone-of-voice without using any slang and becoming too friendly and overly humorous. Rather, the intervention provides users with social cues that, in turn, may elicit corresponding social responses. For example, in each session, one of the agents greets the user at the beginning of the session and says goodbye at the end, as if the intervention was a person. Several sessions also start by reviewing homework from the previous session and end by reminding the user about her homework for the next session. This may develop and build a user narrative with a past, present, and future that may help establish a form of continuity in the relationship. Oftentimes, personal pronouns were used and an active voice was preferred over a passive voice.

### Step 5: Adoption and Implementation

The Norwegian Public Association for Women’s Health [89] is a nonprofit organization that works toward improving women’s living conditions. They have sponsored the development of the intervention, cover its operating costs, and have the ownership and distribution rights to Mamma Mia in Norway. The plan is to implement the intervention in 2 steps: First, in the current version of Mamma Mia, the intervention is marketed directly to pregnant women by advertisements and banners on the Internet, social media, newspapers and magazines, and leaflets in GP’s offices and midwife services at hospitals and well-baby clinics. Expectant mothers can enroll for the intervention from a dedicated website. At enrolment, mothers register their email address and create a password, and start the intervention the following Monday. The intervention is meant to be used individually and is free of charge. An important aspect, in this first step, is also media coverage on television, radio, and the Internet, as well as publication of the research on Mamma Mia in scientific journals and conferences. The latter is particularly important in terms of engaging health personnel and building confidence in the intervention to establish Mamma Mia as part of the basic and supplementary education for health personnel.

The second, long-term step in the implementation of Mamma Mia is to develop guidelines for the implementation of the intervention by GPs, midwives, and PHNs working in well-baby clinics. This entails designing supplementary education for midwives and PHNs who are provided with in-depth knowledge about Mamma Mia and trained in the delivery of the intervention in practice (eg, skills training), which, importantly, will de facto turn Mamma Mia into a guided Internet intervention. This currently ongoing work will be offered as supplementary training at the Centre for Child and Adolescent Mental Health [90] in 2016. It is worthwhile, however, to note that the implementation of any intervention requires more than just training and supervision. The implementation guidelines, which are currently work in progress, will be based on Fixsen and colleagues’ implementation components framework [91]. This means that the implementation guidelines will also specify the formal and informal qualifications required for midwives and PHNs for the supplementary training and supervision. This is referred to as the competency drivers in Fixsen and colleagues’ implementation framework [91]. However, according to their framework, it is equally important to focus on organizational drivers such as evaluation of midwives’ and PHNs’ performance, decision-support systems for quality assurance and improvement of Mamma Mia, administrative support to ensure that leaders, policies, procedures, climate, and other structures are aligned with the needs of midwives and PHNs using Mamma Mia in their practice, and strategies to work with external systems to ensure that necessary resources required to support the sustainability of Mamma Mia over time in well-baby clinics are available (ie, system intervention).

### Step 6: Evaluation

The feasibility of the intervention was demonstrated during piloting [20]. Most participants found Mamma Mia to be acceptable and user friendly, and instilling confidence in the intervention, especially for participants recruited through hospitals and well-baby clinics. In the feasibility study, we also identified several issues that needed improvements, some of which are included in the latest version of Mamma Mia (eg, information about expectations for the postpartum period and the baby’s sleeping patterns). Not all improvements were implemented as they were not feasible at the time (eg, requiring a high level of tailoring that would require extensive re-designing of the program), but remain a part of the long-term plans for the future quality improvement of the intervention.

At the time of this writing, there is an ongoing randomized controlled trial (RCT), which aims to test the effectiveness of Mamma Mia delivered in addition to usual care (for protocol, see [92]). The control group will receive only treatment as usual during pregnancy and maternity care. In Norway, ordinary prenatal and postnatal care includes visits to GP, midwifery services at well-baby clinics and hospitals, and PHNs at well-baby clinics. According to the Norwegian guidelines for prenatal and postnatal care [93,94], this will typically include about 9 consultations during pregnancy and about 10 consultations during the 1st year of postnatal care. We hypothesize that women who receive Mamma Mia will have lower depressive symptoms postpartum than women in the control group, and higher levels of subjective well-being.

In parallel to the RCT, we are interviewing participants who have received the Mamma Mia intervention. The purpose is to assure the quality of the program and contribute strategically to its quality improvement to avoid that the intervention becomes obsolete given today’s rapid technological advances. To this end, we currently use the modified strengths, weaknesses, opportunities, and threats (SWOT) format. The SWOT format does not impose any specific assumptions or themes on the interview nor specify any particular types of answers. Yet, the SWOT format provides a certain structure to the interview and participants’ reflections along 2 dimensions (ie, positive-negative and past-future).

Finally, it becomes important to assess the supplementary training for midwifes and PHNs and implementation of Mamma Mia at well-baby clinics. The supplementary training is considered an important way of strengthening the implementation by making midwifes and PHNs familiar with Mamma Mia and associating it with knowledge or themes, which, according to the national guidelines, midwifes and PHNs should cover during consultations with pregnant and postpartum women and their partners. The supplementary training is also designed so that midwives and PHNs work actively with several of the organizational drivers to embed Mamma Mia into their local well-baby clinic and secure managerial and organizational commitment to the program, as part of the course requirements. This will hopefully help Mamma Mia to become an integrated part of the ordinary health services, and to ensure the dissemination and sustainability of the program.

## Discussion

### Preliminary Findings

The aim of this paper was to provide a comprehensive overview of Mamma Mia and its development using the IM protocol. There are 2 overarching aims in IM: first, it serves as a tool in developing an intervention in a systematic manner. It links the phases of the intervention development to theory and empirical evidence, and thus ensures that decision making is based on sound logic. Second, the IM protocol makes the process of intervention development explicit and transparent. As such, the paper describes a fully automated Internet intervention (Mamma Mia) that is available on personal computers, tablets, and smartphones, and is also platform independent (ie, available on devices from Apple, Google, Windows, etc). The program consists of 3 phases: the first phase, which starts in the 2nd trimester, consists of 11 sessions. It starts in the gestational week 18-24, and ends in the gestational week 40 (ie, estimated due date). The active phase starts when the infant is 2-3 weeks old, and lasts for 6 weeks, with 3 sessions/week. The final phase is the follow-up phase, which consists of 10 sessions spread over an 18-week period. In total, the program consists of 44 sessions disseminated over a period of 11.5 months.

Users of the program progress through the intervention in a tunneled sequence with content centered around topics concerning coping with the transition to parenthood and signs of sadness or depression, engaging with the baby and one’s partner, and engaging in self-caring behaviors. The tunneled design is in accordance with the preparation and psychological reorganization of becoming a mother (and father, [95]), and the topics are based on our needs assessment, which, in turn, shaped the overall goals of the intervention. The overall goals were used to derive the performance objectives which together with its determinants were used to specify the change objectives that guided the development of the intervention program further, in terms of both the selection of theory-based methods and the principles underlying the design of intervention strategies and materials. It describes why and how change methods derived from, for example, couples’ therapy (eg, [96]), positive psychology (eg, [64]), and attachment theory (eg, [71]), were included in the final intervention. As such, it unravels the logic of the development of the current intervention by linking objectives, theories, and actual program materials and activities, and provides a blueprint of the intervention.

The adoption and implementation planning was largely in line with what is prescribed by the IM approach. Potential adopters and implementers of the intervention were included in the development, adoption, and implementation processes from the outset [20], as recommended [24]. However, the actual course of action deviates from the IM approach in that the evaluation of the intervention has focused primarily on the efficacy of the intervention, in terms of behavior change, at the expense of process evaluation. Thus far, this study does not present any evaluation data. Hence, we cannot make any conclusions about its ability to prevent cases of depression, effectiveness in reducing depressive symptoms, or increasing subjective well-being. However, the transparency of the intervention content may assist with interpreting results from upcoming studies, anticipate any strengths and weaknesses with the intervention, and make potential changes or improvements that may enhance its effectiveness.

### Generalizability

Mapping the development and contents of an intervention, as in this study, is useful and serves several purposes; first, it allows intervention designers to faithfully replicate effective interventions or attempt to design interventions that are even more effective. Second, it allows researchers to identify mechanisms or techniques contributing to intervention effectiveness (ie, *why* and *how* they work) and extend the evaluation of an intervention to, for example, other populations or settings (eg, for *whom* they work). Third, it may assist educational institutions and other agencies in developing training and implementation strategies for the intervention. Finally, health personnel may more easily determine whether interventions such as Mamma Mia may be an appropriate intervention for their practice and users. Therefore, we argue that this paper illustrates why such post hoc descriptions are useful and comply with the call for transparency and thorough descriptions of interventions that had been noted by numerous authors (see eg, [97-101]).

Systematic and comprehensive descriptions of interventions are necessary to ensure that we can describe aspects of interventions that are generalizable and identify determinants used to achieve change and how the use of theory can be translated into practical strategies. This is necessary to promote and deepen our understanding of the development of complex interventions and move on from general remarks and conclusions such as “...more extensive use of theory was associated with increases in effect size...” [102] to a higher level of specificity. More research is needed to identify exactly which theoretical frameworks and practical strategies work best. However, this is the stepping stone that can help advance the accumulation of scientific knowledge in eHealth and prevent “type III error,” that is, rejection of an intervention’s effectiveness because the intervention itself is poorly designed or implemented [103]. Furthermore, this may help advance the usefulness of systematic reviews, which identify, critically appraise, and synthesize relevant research, but this depends on the quality of the descriptions of the available interventions under investigation. Therefore, we argue that researchers should routinely publish peer-reviewed intervention protocols separately prior to publishing results from evaluation studies (ie, similar to registration of trial protocols in registries such as ClinicalTrials.gov or International Standard Randomised Controlled Trial Number). This will help get a fuller understanding of what exactly is being evaluated, compare treatments against each other, and learn from previous development efforts.

### Limitations

Based on the IM protocol, this paper has 1 key limitation: it was applied in a post hoc manner. Hence, the development process described in this paper deviates somewhat from the IM protocol. Nevertheless, it is important to note that the IM framework influenced the development of Mamma Mia, which makes a post hoc analysis both feasible and informative. For instance, a long-term perspective in this project made it possible for us to perform studies on the determinants of PPD in Norwegian mothers [33,34,104], which is a key step in intervention development according to IM. As previous studies illustrate, a retrospective IM-based analysis can be useful as it may point toward weaknesses in the intervention development process and the intervention itself [27,28], thereby providing information on any potential threats and issues that may arise mainly during the implementation and evaluation.

In a sense, this may also be one of the strengths of applying IM analysis post hoc and highlights why it is better to perform IM after the development of an intervention, rather than never. For example, dropout rates in Internet interventions tend to be high [105,106]. Thus, designing an intervention consisting of 44 sessions over a period of almost 1 year adds numerous opportunities for discontinuation. Although many pregnant and postpartum women may be “eHealth information seekers,” it cannot be expected that this alone will sustain engagement with the intervention for extended periods, without any human involvement. By contrast, the lack of segmentation of the target population may prove to be more or less acceptable to certain subgroups. The intervention targets both young, first-time mothers who may feel more insecure and in need of support, and older, multiparous women who may have a greater sense of security and confidence in their parenting abilities. Mamma Mia may also be more appropriate for women living with a partner than single mothers and for women without complications. These factors may be used to guide future quality improvement of the intervention; however, currently, they also pose a threat to intervention effectiveness.

### Conclusion

In the development of any type of intervention, intervention designers translate theory into practice. However, there are many steps during development where the theoretical methods believed to be effective can be “lost in translation.” By applying the IM protocol to Mamma Mia, we have described the rationale and contents of the intervention, and opened up the black box for intervention designers, researchers, educational institutions, and health personnel working with perinatal mental health. The IM analysis of Mamma Mia helps to make clear decisions regarding intervention development from theory, empirical findings, and practical strategies that may contribute to its overall effectiveness. It also makes transparent some of the potential pitfalls such as its duration and lack of segmentation that may jeopardize intervention effectiveness. Overall, this paper adds to the plea for systematic reporting of intervention protocols that document the design and development of interventions, and accumulation of knowledge about interventions in the field of eHealth.

## Appendix

Multimedia Appendix 1 Intervention screenshots from Mamma Mia.

Multimedia Appendix 2 Transcripts from program materials.

## Abbreviations

CoS: circle of security
EPDS: Edinburgh Postnatal Depression Scale
GP: general physician
IM: intervention mapping
MCT: metacognitive therapy
NBO: newborn behavioral observation
PHNs: public health nurses
PPD: postpartum depression
PPIs: positive psychology interventions
SWOT: strengths, weaknesses, opportunities, and threats

## References

[1] Munk-Olsen T, Laursen T, Pedersen C, Mors O, Mortensen P (2006). New parents and mental disorders: A population-based register study. JAMA.

[2] O’Hara M, Swain A (1996). Rates and risk of postpartum depression: A meta-analysis. Int Rev Psychiatry.

[3] Gavin NI, Gaynes BN, Lohr KN, Meltzer-Brody S, Gartlehner G, Swinson T (2005). Perinatal depression: A systematic review of prevalence and incidence. Obstet Gynecol.

[4] Musters C, McDonald E, Jones I (2008). Management of postnatal depression. BMJ.

[5] Banti S, Mauri M, Oppo A, Borri C, Rambelli C, Ramacciotti D, Montagnani MS, Camilleri V, Cortopassi S, Rucci P, Cassano GB (2011). From the third month of pregnancy to 1 year postpartum. Prevalence, incidence, recurrence, and new onset of depression. Results from the perinatal depression-research & screening unit study. Compr Psychiatry.

[6] Lovejoy M, Graczyk P, O'Hare E, Neuman G (2000). Maternal depression and parenting behavior: A meta-analytic review. Clin Psychol Rev.

[7] Goodman SH, Rouse MH, Connell AM, Broth MR, Hall CM, Heyward D (2011). Maternal depression and child psychopathology: A meta-analytic review. Clin Child Fam Psychol Rev.

[8] Paulson J, Bazemore S (2010). Prenatal and postpartum depression in fathers and its association with maternal depression: A meta-analysis. JAMA.

[9] Cuijpers P, Weitz E, Karyotaki E, Garber J, Andersson G (2015). The effects of psychological treatment of maternal depression on children and parental functioning: A meta-analysis. Eur Child Adolesc Psychiatry.

[10] Flynn HA, Blow FC, Marcus SM (2006). Rates and predictors of depression treatment among pregnant women in hospital-affiliated obstetrics practices. Gen Hosp Psychiatry.

[11] Bauer A, Parsonage M, Knapp M, Iemmi V, Adelaja B (2014). The Costs of Perinatal Mental Health Problems.

[12] Larsson M (2009). A descriptive study of the use of the Internet by women seeking pregnancy-related information. Midwifery.

[13] (2015). Bruk av IKT i Husholdningene [Use of Information and Communication Technology in Households].

[14] Fox S (2011). Health Topics.

[15] Maloni JA, Przeworski A, Damato EG (2013). Web recruitment and internet use and preferences reported by women with postpartum depression after pregnancy complications. Arch Psychiatr Nurs.

[16] Danaher BG, Milgrom J, Seeley JR, Stuart S, Schembri C, Tyler MS, Ericksen J, Lester W, Gemmill AW, Kosty DB, Lewinsohn P (2013). MomMoodBooster web-based intervention for postpartum depression: Feasibility trial results. J Med Internet Res.

[17] Danaher BG, Milgrom J, Seeley JR, Stuart S, Schembri C, Tyler MS, Ericksen J, Lester W, Gemmill AW, Lewinsohn P (2012). Web-based intervention for postpartum depression: Formative research and design of the MomMoodBooster program. JMIR Res Protoc.

[18] Salonen AH, Pridham KF, Brown RL, Kaunonen M (2014). Impact of an internet-based intervention on Finnish mothers' perceptions of parenting satisfaction, infant centrality and depressive symptoms during the postpartum year. Midwifery.

[19] Logsdon MC, Barone M, Lynch T, Robertson A, Myers J, Morrison D, York S, Gregg J (2013). Testing of a prototype web based intervention for adolescent mothers on postpartum depression. Appl Nurs Res.

[20] Haga S, Drozd F, Brendryen H, Slinning K (2013). Mamma Mia: A feasibility study of a web-based intervention to reduce the risk of postpartum depression and enhance subjective well-being. JMIR Res Protoc.

[21] Sheeber LB, Seeley JR, Feil EG, Davis B, Sorensen E, Kosty DB, Lewinsohn PM (2012). Development and pilot evaluation of an Internet-facilitated cognitive-behavioral intervention for maternal depression. J Consult Clin Psychol.

[22] O'Mahen HA, Woodford J, McGinley J, Warren FC, Richards DA, Lynch TR, Taylor RS (2013). Internet-based behavioral activation—Treatment for postnatal depression (Netmums): A randomized controlled trial. J Affect Disord.

[23] O'Mahen HA, Richards DA, Woodford J, Wilkinson E, McGinley J, Taylor RS, Warren FC (2014). Netmums: A phase II randomized controlled trial of a guided Internet behavioural activation treatment for postpartum depression. Psychol Med.

[24] Bartholomew L, Parcel G, Kok G, Gottlieb N (2006). Planning Health Promotion Programs: An Intervention Mapping Approach. 2nd ed.

[25] Bartholomew L, Mullen P (2011). Five roles for using theory and evidence in the design and testing of behavior change interventions. J Public Health Dent.

[26] Kok G, Mesters I (2011). Getting inside the black box of health promotion programmes using intervention mapping. Chronic Illn.

[27] Brendryen H, Kraft P, Schaalma H (2010). Looking inside the black box: Using intervention mapping to describe the development of the automated smoking cessation intervention happy ending. J Smok Cessat.

[28] Brendryen H, Johansen A, Nesvåg S, Kok G, Duckert F (2013). Constructing a theory- and evidence-based treatment rationale for complex eHealth interventions: Development of an online alcohol intervention using an intervention mapping approach. JMIR Res Protoc.

[29] Whitton A, Warner R, Appleby L (1996). The pathway to care in post-natal depression: Women's attitudes to post-natal depression and its treatment. Br J Gen Pract.

[30] Buist A, Bilszta J, Barnett B, Milgrom J, Ericksen J, Condon J, Hayes B, Brooks J (2005). Recognition and management of perinatal depression in general practice—A survey of GPs and postnatal women. Aust Fam Physician.

[31] Dennis C, Chung-Lee L (2006). Postpartum depression help-seeking barriers and maternal treatment preferences: A qualitative systematic review. Birth.

[32] Howard LM, Molyneaux E, Dennis C, Rochat T, Stein A, Milgrom J (2014). Non-psychotic mental disorders in the perinatal period. Lancet.

[33] Haga SM, Ulleberg P, Slinning K, Kraft P, Steen TB, Staff A (2012). A longitudinal study of postpartum depressive symptoms: Multilevel growth curve analyses of emotion regulation strategies, breastfeeding self-efficacy, and social support. Arch Womens Ment Health.

[34] Haga SM, Lynne A, Slinning K, Kraft P (2012). A qualitative study of depressive symptoms and well-being among first-time mothers. Scand J Caring Sci.

[35] Røsand G-MB, Slinning K, Eberhard-Gran M, Røysamb E, Tambs K (2012). The buffering effect of relationship satisfaction on emotional distress in couples. BMC Public Health.

[36] Røsand G-MB, Slinning K, Eberhard-Gran M, Røysamb E, Tambs K (2011). Partner relationship satisfaction and maternal emotional distress in early pregnancy. BMC Public Health.

[37] Proulx C, Helms H, Buehler C (2007). Marital quality and personal well-being: A meta-analysis. J Marriage Fam.

[38] Luhmann M, Hofmann W, Eid M, Lucas RE (2012). Subjective well-being and adaptation to life events: A meta-analysis. J Pers Soc Psychol.

[39] Dyrdal GM, Røysamb E, Nes RB, Vittersø J (2011). Can a happy relationship predict a happy life? A population-based study of maternal well-being during the life transition of pregnancy, infancy, and toddlerhood. J Happiness Stud.

[40] Weinberg M, Tronick E (1998). Emotional characteristics of infants associated with maternal depression and anxiety. Pediatrics.

[41] Field T (2010). Postpartum depression effects on early interactions, parenting, and safety practices: A review. Infant Behav Dev.

[42] Barker ED, Jaffee SR, Uher R, Maughan B (2011). The contribution of prenatal and postnatal maternal anxiety and depression to child maladjustment. Depress Anxiety.

[43] Velders FP, Dieleman G, Henrichs J, Jaddoe VWV, Hofman A, Verhulst FC, Hudziak JJ, Tiemeier H (2011). Prenatal and postnatal psychological symptoms of parents and family functioning: The impact on child emotional and behavioural problems. Eur Child Adolesc Psychiatry.

[44] Leis JA, Heron J, Stuart EA, Mendelson T (2014). Associations between maternal mental health and child emotional and behavioral problems: Does prenatal mental health matter?. J Abnorm Child Psychol.

[45] Korhonen M, Luoma I, Salmelin R, Tamminen T (2012). A longitudinal study of maternal prenatal, postnatal and concurrent depressive symptoms and adolescent well-being. J Affect Disord.

[46] Pearson RM, Evans J, Kounali D, Lewis G, Heron J, Ramchandani PG, O'Connor TG, Stein A (2013). Maternal depression during pregnancy and the postnatal period: Risks and possible mechanisms for offspring depression at age 18 years. JAMA Psychiatry.

[47] Atkinson L, Paglia A, Coolbear J, Niccols A, Parker K, Guger S (2000). Attachment security: A meta-analysis of maternal mental health correlates. Clin Psychol Rev.

[48] Hayes LJ, Goodman SH, Carlson E (2013). Maternal antenatal depression and infant disorganized attachment at 12 months. Attach Hum Dev.

[49] Benoit D, Parker KCH, Zeanah CH (1997). Mothers' representations of their infants assessed prenatally: Stability and association with infants' attachment classifications. J Child Psychol Psychiatry.

[50] Glaze R, Cox J (1991). Validation of a computerised version of the 10-item (self-rating) Edinburgh Postnatal Depression Scale. J Affect Disord.

[51] Spek V, Nyklícek I, Cuijpers P, Pop V (2008). Internet administration of the Edinburgh Depression Scale. J Affect Disord.

[52] Larun L, Fønhus M, Håvelsrud K, Brurberg K, Reinar L (2013). Depresjonsscreening av Gravide og Barselkvinner [Depression Screening of Pregnant and Postpartum Women].

[53] Morrell CJ, Warner R, Slade P, Dixon S, Walters S, Paley G, Brugha T (2009). Psychological interventions for postnatal depression: Cluster randomised trial and economic evaluation. The PoNDER trial. Health Technol Assess.

[54] Wells A (2009). Metacognitive Therapy for Anxiety and Depression.

[55] Bevan D, Wittkowski A, Wells A (2013). A multiple-baseline study of the effects associated with metacognitive therapy in postpartum depression. J Midwifery Womens Health.

[56] Brugha TS, Morrell CJ, Slade P, Walters SJ (2011). Universal prevention of depression in women postnatally: Cluster randomized trial evidence in primary care. Psychol Med.

[57] MacArthur C, Winter H, Bick D, Knowles H, Lilford R, Henderson C, Lancashire RJ, Braunholtz DA, Gee H (2002). Effects of redesigned community postnatal care on womens' health 4 months after birth: A cluster randomised controlled trial. Lancet.

[58] Drozd F, Mork L, Nielsen B, Raeder S, Bjørkli CA (2014). Better days—A randomized controlled trial of an internet-based positive psychology intervention. J Posit Psychol.

[59] Schueller SM, Parks AC (2012). Disseminating self-help: Positive psychology exercises in an online trial. J Med Internet Res.

[60] Parks AC, Della Porta MD, Pierce RS, Zilca R, Lyubomirsky S (2012). Pursuing happiness in everyday life: The characteristics and behaviors of online happiness seekers. Emotion.

[61] Chiesa A, Serretti A (2009). Mindfulness-based stress reduction for stress management in healthy people: A review and meta-analysis. J Altern Complement Med.

[62] Boehm J, Lyubomirsky S, Sheldon K (2011). A longitudinal experimental study comparing the effectiveness of happiness-enhancing strategies in Anglo Americans and Asian Americans. Cogn Emot.

[63] Sheldon K, Boehm J, Lyubomirsky S, Boniwell I, David S (2012). Variety is the spice of happiness: The hedonic adaptation prevention (HAP) model. Oxford Handbook of Happiness.

[64] Lyubomirsky S (2007). The How of Happiness: A Scientific Approach to Getting the Life You Want.

[65] Pennebaker J, Chung C, Friedman HS (2011). Expressive writing: Connections to physical mental health. Oxford Handbook of Health Psychology. Vol 7.

[66] Gottman J, Silver N (1999). The Seven Principles of Making Marriage Work.

[67] Gottman JM, Gottman JS, Gurman AS (2008). Gottman method couple therapy. Clinical Handbook of Couple Therapy. 4th ed.

[68] Braithwaite SR, Fincham FD (2009). A randomized clinical trial of a computer based preventive intervention: Replication and extension of ePREP. J Fam Psychol.

[69] Rosenberg M (2003). Nonviolent Communication: A Language of Life. 2nd ed.

[70] Nugent J, Keefer C, Minear S, Johnson L, Blanchard Y (2007). Understanding Newborn Behaviorearly Relationships: The Newborn Behavioral Observations (NBO) System Handbook.

[71] Powell B, Cooper G, Hoffman K, Marvin R (2014). The Circle of Security Intervention: Enhancing Attachment in Early Parent-Child Relationships.

[72] Cohen N, Muir E, Lojkasek M, Johnson SM, Whiffen VE (2006). The first couple: Using watch, wait, wonder to change troubled infant-mother relationships. Attachment Processes in Couple and Family Therapy.

[73] (2014). Norsk Mediebarometer [Norwegian Media Barometer, 2013].

[74] (2015). Fødte, 2014 [Childbirths, 2014].

[75] Danaher B, McKay H, Seeley J (2005). The information architecture of behavior change websites. J Med Internet Res.

[76] Crutzen R, Cyr D, de Vries NK (2012). The role of user control in adherence to and knowledge gained from a website: Randomized comparison between a tunneled version and a freedom-of-choice version. J Med Internet Res.

[77] Mayer R, Moreno R (2003). Nine ways to reduce cognitive load in multimedia learning. Educ Psychol.

[78] Drozd F (2013). Treatment Effects and Consumer Perceptions of Web-Based Interventions [Doctoral Thesis].

[79] Lehto T, Oinas-Kukkonen H, Drozd F (2012). Factors affecting perceived persuasiveness of a behavior change support system. http://aisel.aisnet.org/icis2012/proceedings/HumanBehavior/18/.

[80] Brendryen H, Drozd F, Kraft P (2008). A digital smoking cessation program delivered through internet and cell phone without nicotine replacement (happy ending): Randomized controlled trial. J Med Internet Res.

[81] Brendryen H, Lund IO, Johansen AB, Riksheim M, Nesvåg S, Duckert F (2014). Balance—A pragmatic randomized controlled trial of an online intensive self-help alcohol intervention. Addiction.

[82] (2015). Changetech.

[83] Richardson R, Richards DA, Barkham M (2010). Self-help books for people with depression: The role of the therapeutic relationship. Behav Cogn Psychother.

[84] Barazzone N, Cavanagh K, Richards DA (2012). Computerized cognitive behavioural therapy and the therapeutic alliance: A qualitative enquiry. Br J Clin Psychol.

[85] Bickmore T, Gruber A, Picard R (2005). Establishing the computer-patient working alliance in automated health behavior change interventions. Patient Educ Couns.

[86] Bickmore T, Schulman D, Yin L (2010). Maintaining engagement in long-term interventions with relational agents. Appl Artif Intell.

[87] Consolvo S, Klasnja P, McDonald D, Avrahami D, Froehlich J, LeGrand L, Libby R, Mosher K, Landay JA (2008). Flowers or a robot army?: Encouraging awareness and activity with personal, mobile displays. Proceedings of the 10th International Conference on Ubiquitous Computing.

[88] Kennedy C, Powell J, Payne T, Ainsworth J, Boyd A, Buchan I (2012). Active assistance technology for health-related behavior change: An interdisciplinary review. J Med Internet Res.

[89] (2015). Norske Kvinners Sanitetsforening [Norwegian Women’s Public Health Association].

[90] (2015). Regionsenter for Barn og Unges Psykiske Helse, Helseregion Øst og Sør [Centre for Child and Adolescent Mental Health].

[91] Fixsen D, Naoom S, Blase K, Friedman R, Wallace F (2005). Implementation Research: A Synthesis of the Literature.

[92] Drozd F (2013). A web-based program for depressive symptoms and life satisfaction during pregnancy and after childbirth [ISRCTN91808706].

[93] Norwegian Directorate for Health and Social Affairs (2005). Retningslinjer for Svangerskapsomsorgen [Guidelines for Prenatal Care].

[94] Norwegian Directorate of Health (2013). Nytt liv og Trygg Barseltid for Familien. Retningslinje for Barselomsorgen [A New Life and a Safe Puerperium for the Family. Guidelines for Postnatal Care].

[95] Birss S, Nugent JK, Keefer CH, Minear S, Johnson LC, Blanchard Y (2007). Transition to parenthood: Promoting the parent-infant relationship. Understanding Newborn Behavior and Early Relationships: The Newborn Behavioral Observations (NBO) System Handbook.

[96] Gottman JM, Gottman JS (2006). 10 Lessons to Transform Your Marriage.

[97] Schaalma H, Kok G (2009). Decoding health education interventions: The times are a-changin'. Psychol Health.

[98] Michie S, Abraham C (2008). Advancing the science of behaviour change: A plea for scientific reporting. Addiction.

[99] Dombrowski SU, Sniehotta FF, Avenell A, Coyne JC (2007). Towards a cumulative science of behaviour change: Do current conduct and reporting of behavioural interventions fall short of best practice?. Psychol Health.

[100] Riley BL, MacDonald J, Mansi O, Kothari A, Kurtz D, von Tettenborn LI, Edwards NC (2008). Is reporting on interventions a weak link in understanding how and why they work? A preliminary exploration using community heart health exemplars. Implement Sci.

[101] Hoffmann TC, Glasziou PP, Boutron I, Milne R, Perera R, Moher D, Altman DG, Barbour V, Macdonald H, Johnston M, Lamb SE, Dixon-Woods M, McCulloch P, Wyatt JC, Chan A, Michie S (2014). Better reporting of interventions: Template for intervention description and replication (TIDieR) checklist and guide. BMJ.

[102] Webb T, Joseph J, Yardley L, Michie S (2010). Using the internet to promote health behavior change: A systematic review and meta-analysis of the impact of theoretical basis, use of behavior change techniques, and mode of delivery on efficacy. J Med Internet Res.

[103] Green J (2000). The role of theory in evidence-based health promotion practice. Health Educ Res.

[104] Haga S (2011). Identifying risk factors for postpartum depressive symptoms: The importance of social support, self-efficacy, and emotion regulation [doctoral thesis].

[105] Mathieu E, McGeechan K, Barratt A, Herbert R (2013). Internet-based randomized controlled trials: A systematic review. J Am Med Inform Assoc.

[106] Farvolden P, Denisoff E, Selby P, Bagby RM, Rudy L (2005). Usage and longitudinal effectiveness of a Web-based self-help cognitive behavioral therapy program for panic disorder. J Med Internet Res.

