# Supplementary material for: An Internet-Based Intervention (Mamma Mia) for Postpartum Depression: Mapping the Development from Theory to Practice
Source: JMIR Res Protoc. 2015 Oct 12;4(4):e120. doi: 10.2196/resprot.4858 (PMC4704906; doi:10.2196/resprot.4858)
Supplement: Multimedia Appendix 1 [file resprot_v4i4e120_app1.pdf]

Multimedia Appendix 1: Screenshots*

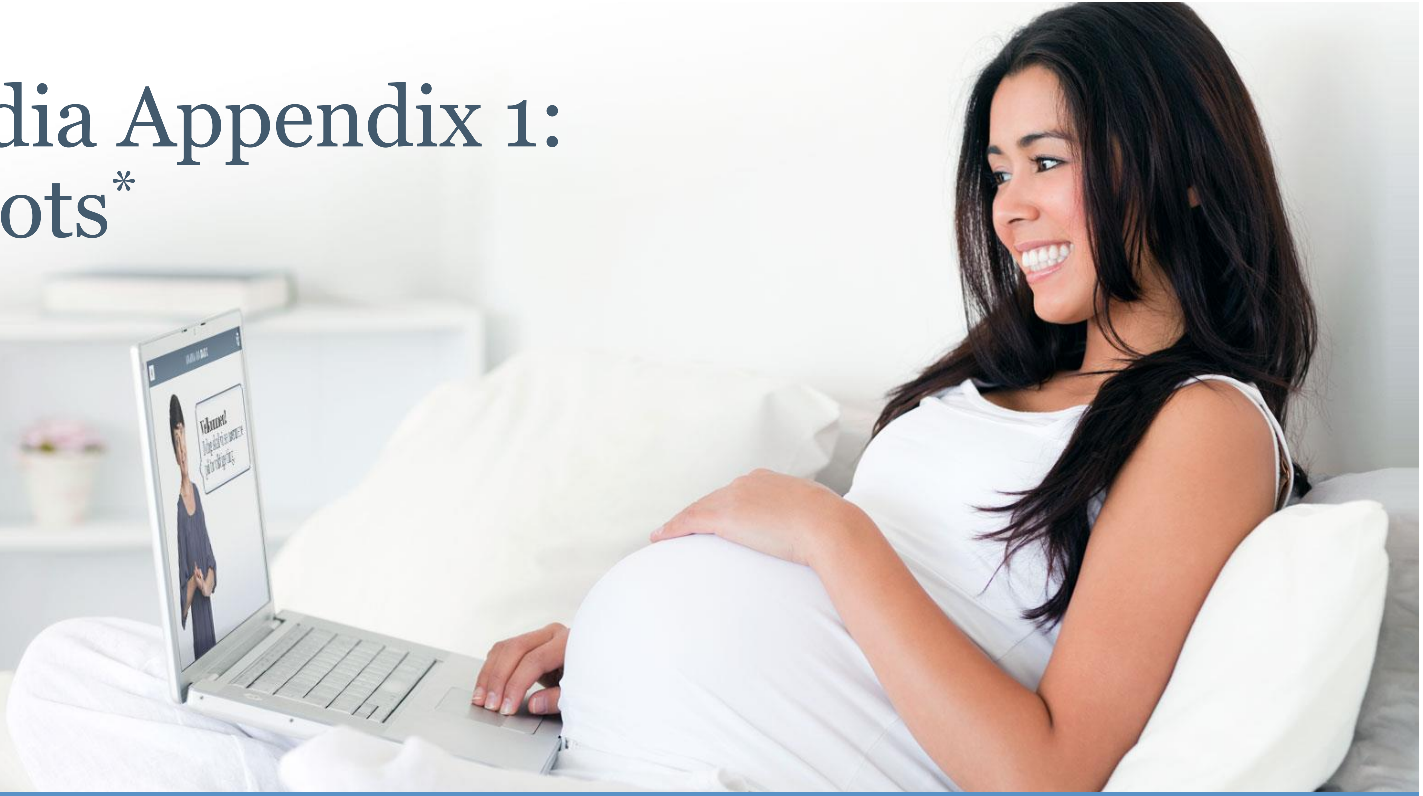

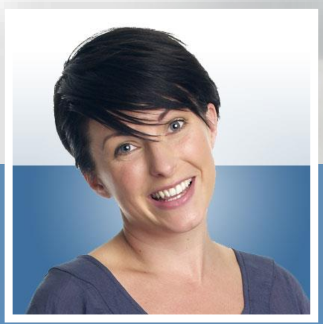

Mamma Mia

* In this presentation, some screenshots are taken out of their context and may not fully represent Mamma Mia.

Edinburgh Postnatal Depression Scale

Metacognitive Therapy

Subjective Well-Being

Couple Relationship

Parental Sensitivity

Screening for depressive symptoms

Presenting the rationale for assessing depressive symptoms

In a friendly and empathic tone-of-voice, using a guide to give the program a 'human touch'

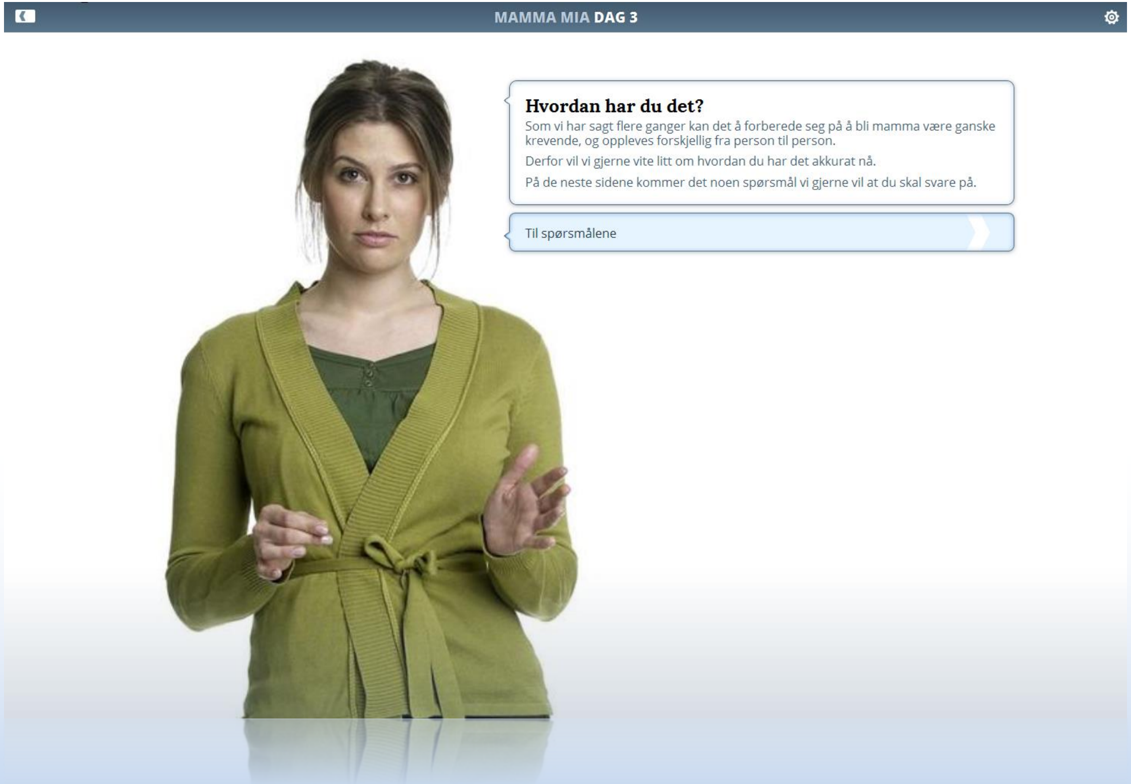

Screening for depressive symptoms

Question from the Edinburgh Postnatal Depression Scale:

‘I have been able to laugh and see the funny side of things’

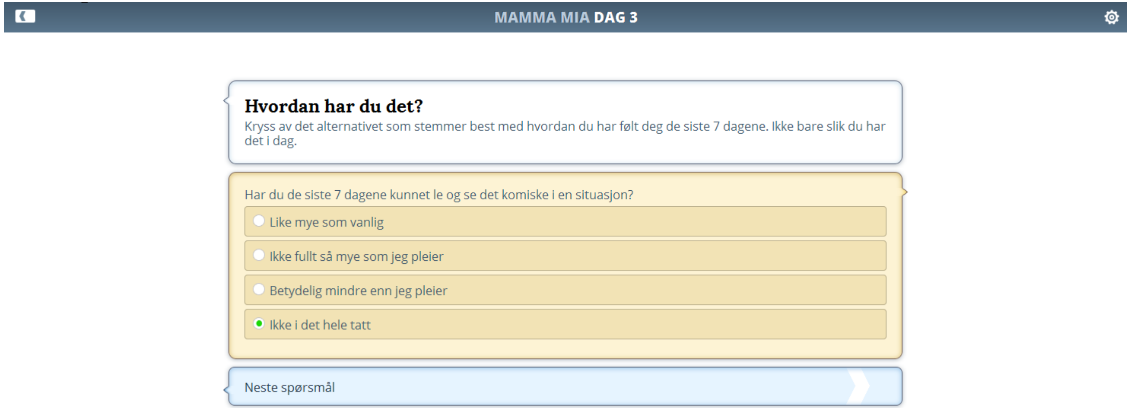

Screening for depressive symptoms

Question from the Edinburgh Postnatal Depression Scale:

‘I have looked forward with enjoyment to things’

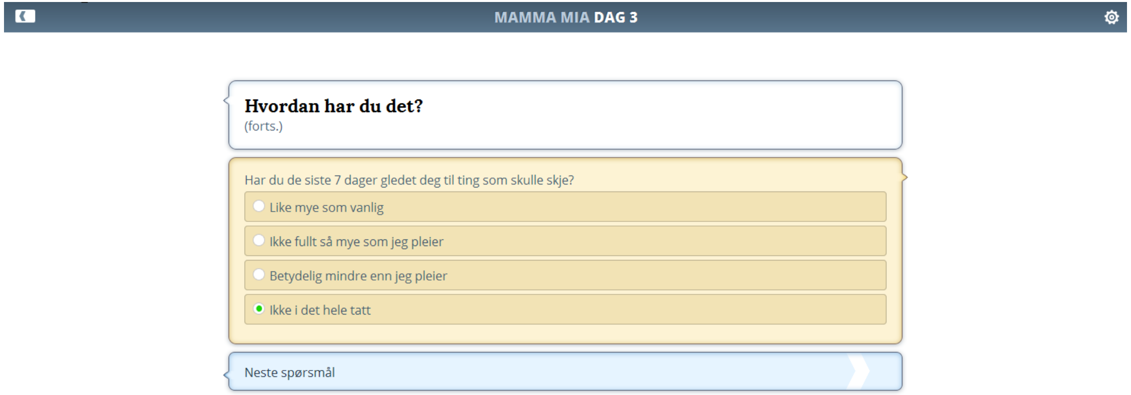

Screening for depressive symptoms

Question from the Edinburgh Postnatal Depression Scale:

‘I have felt scared or panicky for no very good reason’

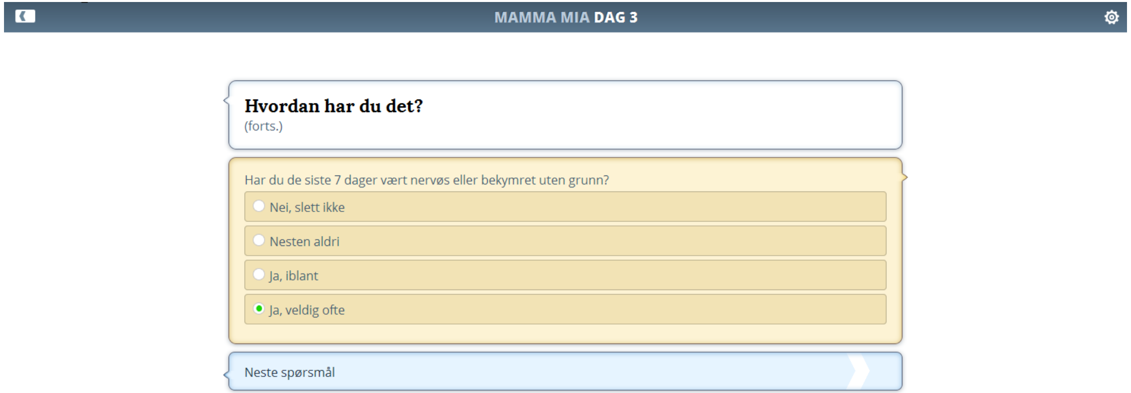

Screening for depressive symptoms

EPDS-score below 10 results in supportive feedback

Explain why it is important to re-assess depressive symptoms

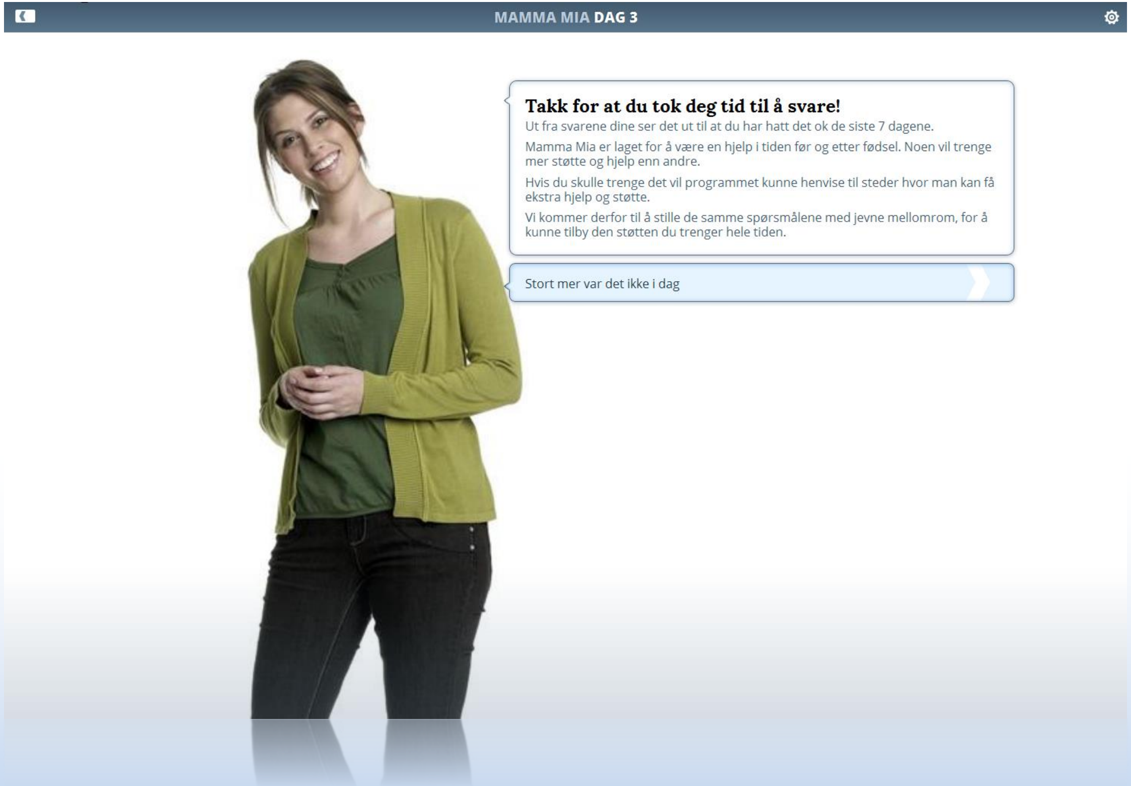

- Edinburgh Postnatal Depression Scale
- **Metacognitive Therapy**
- Subjective Well-Being
- Couple Relationship
- Parental Sensitivity

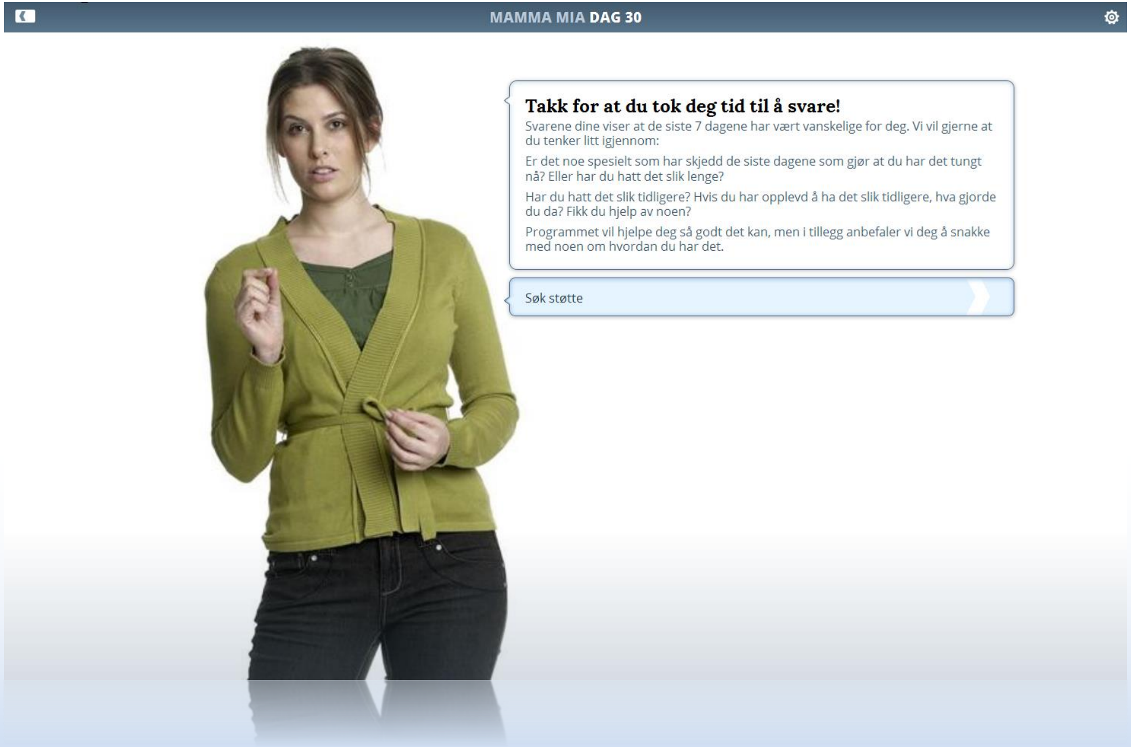

Help and support for depressive symptoms

EPDS-score of 10 or higher results in supportive feedback

Self-reflection is encouraged:

‘Is there anything special that has happened lately or have felt this way for quite a while?’

Mamma Mia

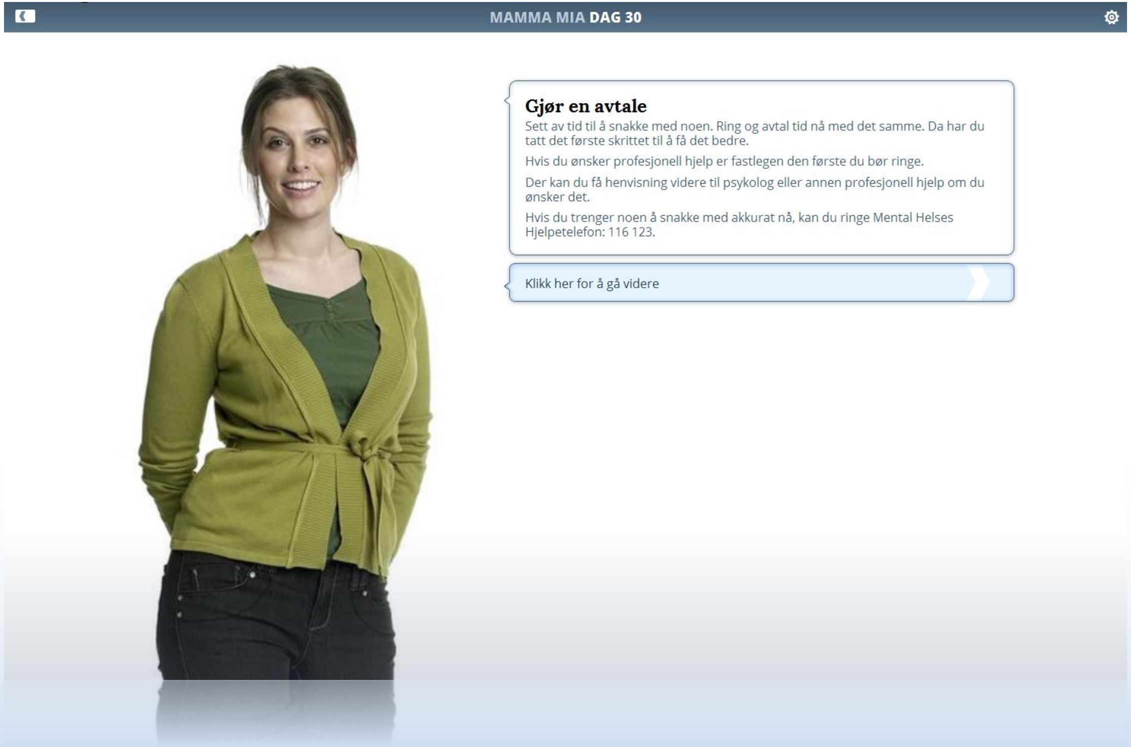

Help and support for depressive symptoms

Encourage help-seeking, e.g., via the general physician

Call a mental health hotline if you need someone to talk to right away

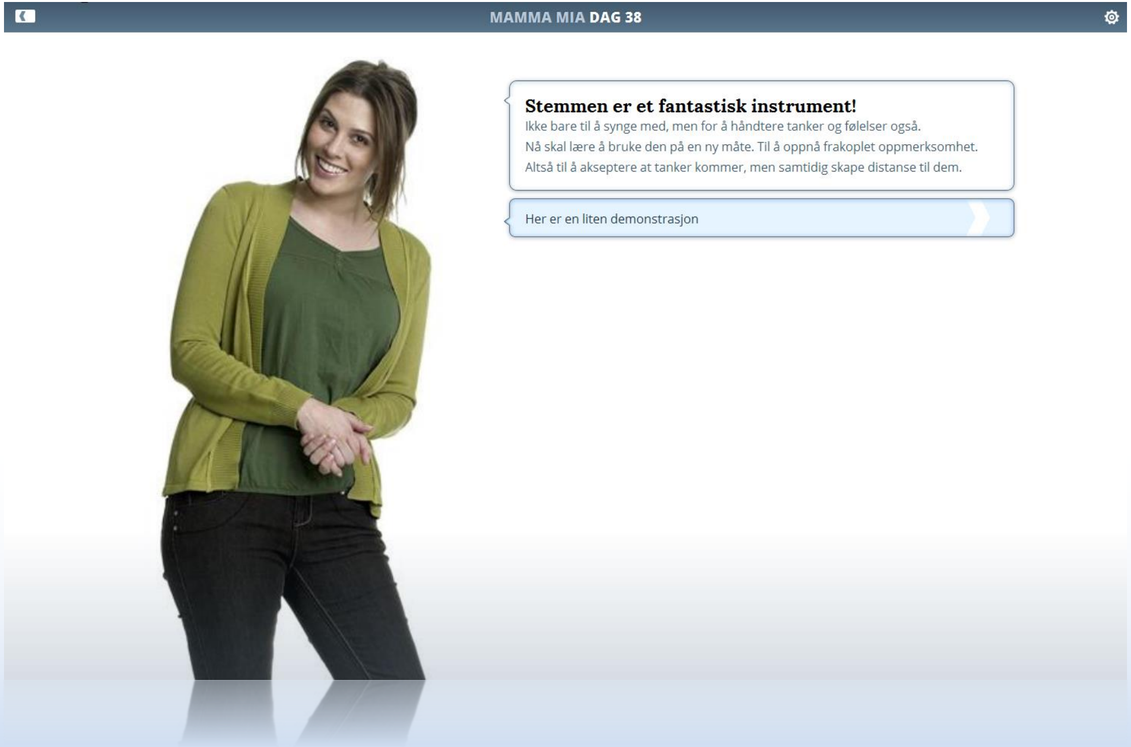

Help and support for depressive symptoms

Mamma Mia provides immediate online help and support

Exercises to support psychological flexibility

Mamma Mia

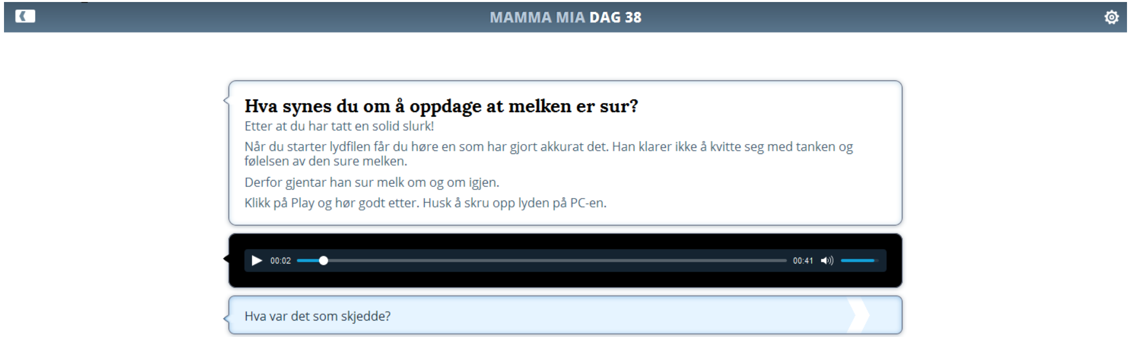

Help and support for depressive symptoms

Demonstration of a technique for cognitive defusion

Promoting defusion in the presence of negative thoughts and feelings

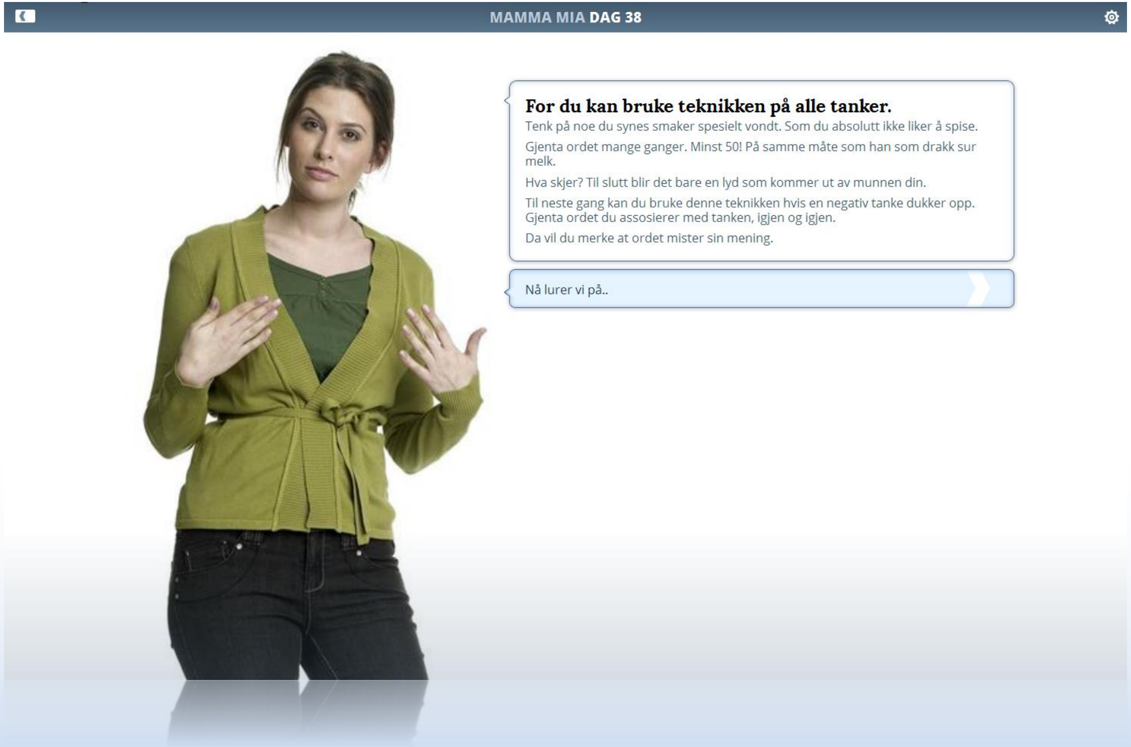

Help and support for depressive symptoms

Translating defusion to everyday life

Encourage to give it a try and use it the next time a negative thought occurs.

Edinburgh Postnatal Depression Scale → Metacognitive therapy → **Subjective Well-Being** → Couple Relationship → Parental Sensitivity

Prescriptions for the good life – Stress management

Stress and common reactions to stress explained

Expecting a baby can be a stressful event

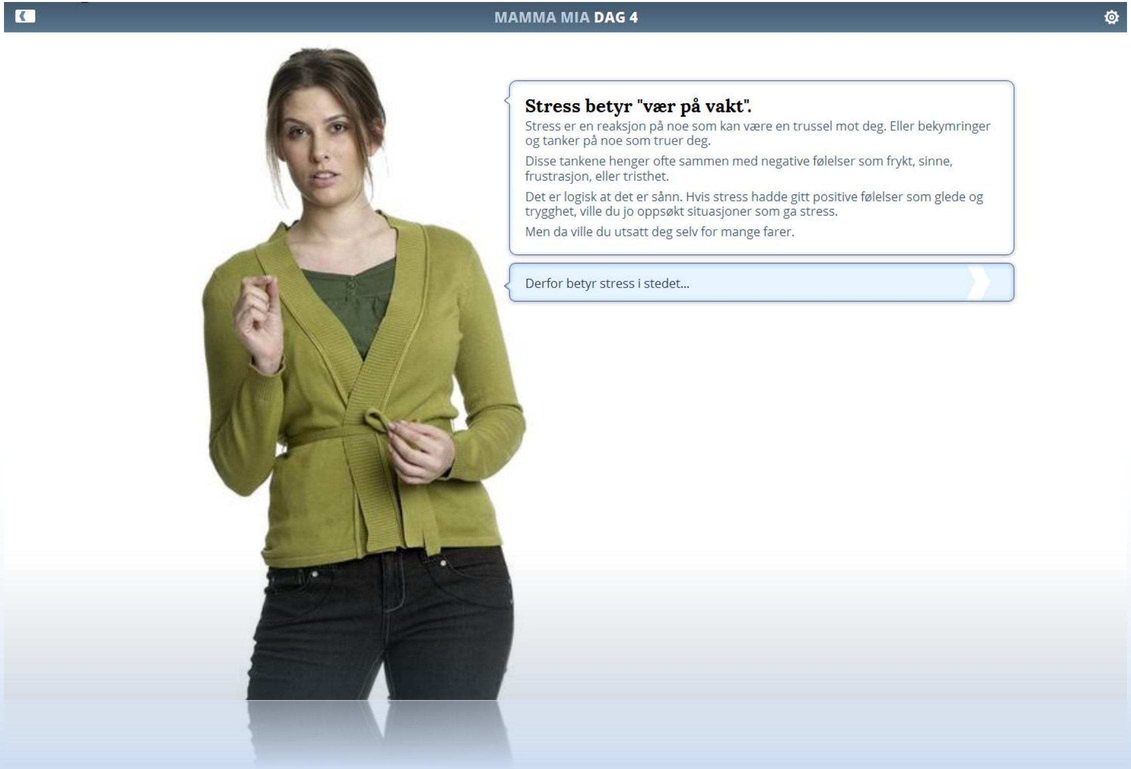

Prescriptions for the good life – Stress management

Instilling confidence in stress management techniques

Introducing mindfulness

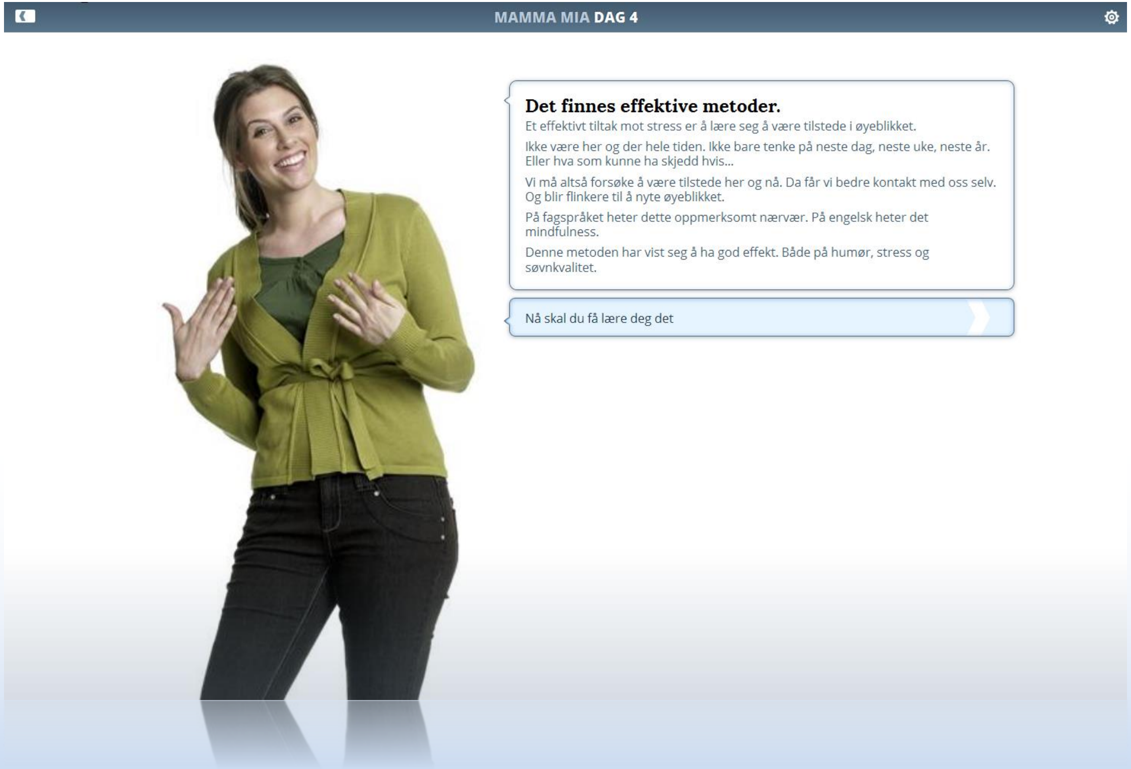

Prescriptions for the good life – Stress management

Audio-guided mindfulness instructions

Downloadable audio file for homework and practice

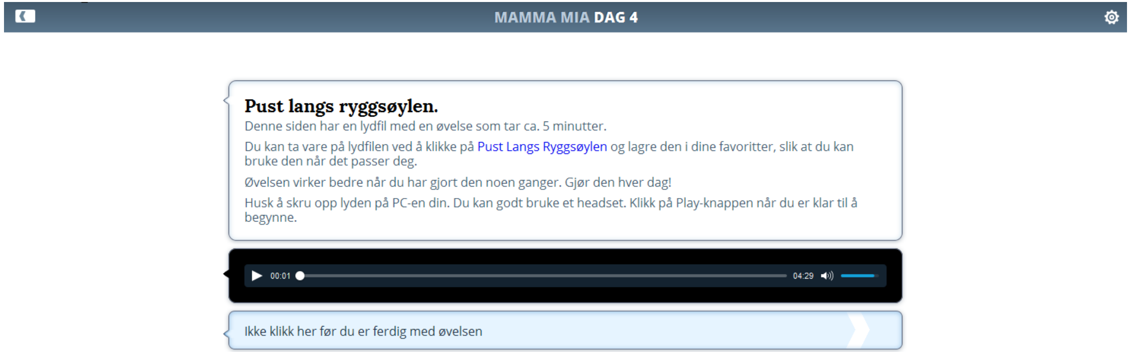

Edinburgh Postnatal Depression Scale

Metacognitive therapy

Subjective Well-Being

Couple Relationship

Parental Sensitivity

Mamma Mia

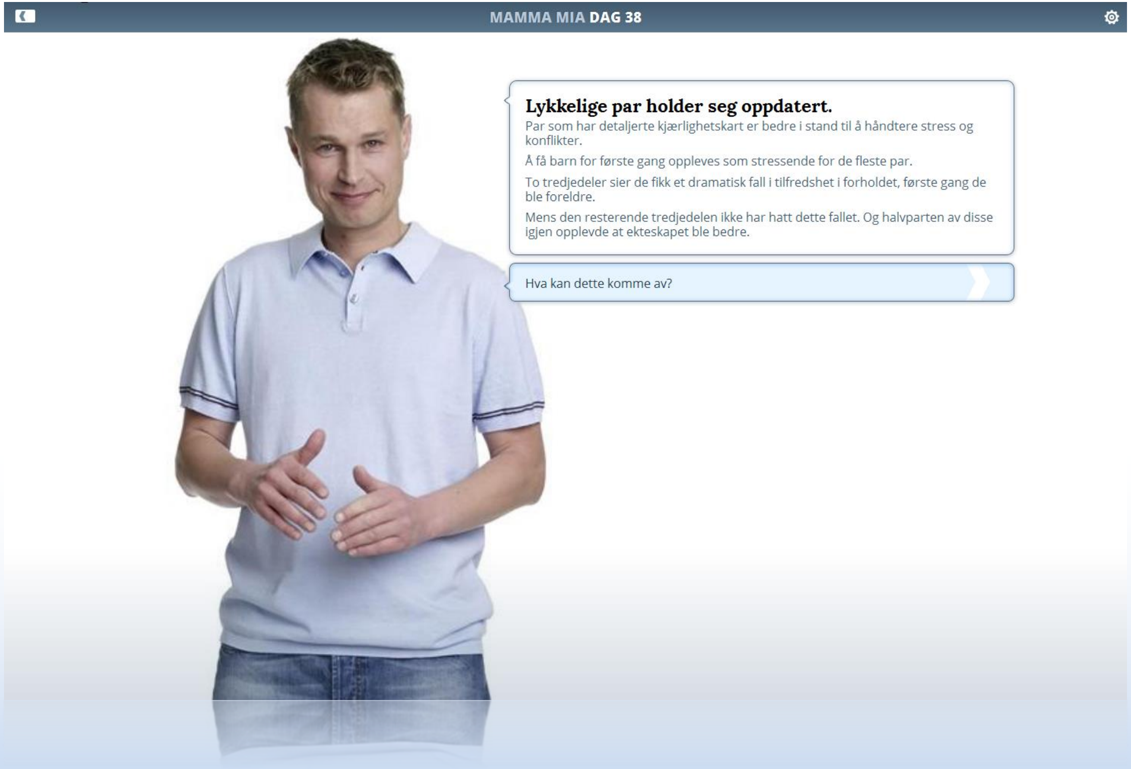

Building closeness and partner intimacy

Expecting a baby can be distressing for the couple

Buffering effects of relationship satisfaction explained

Mamma Mia

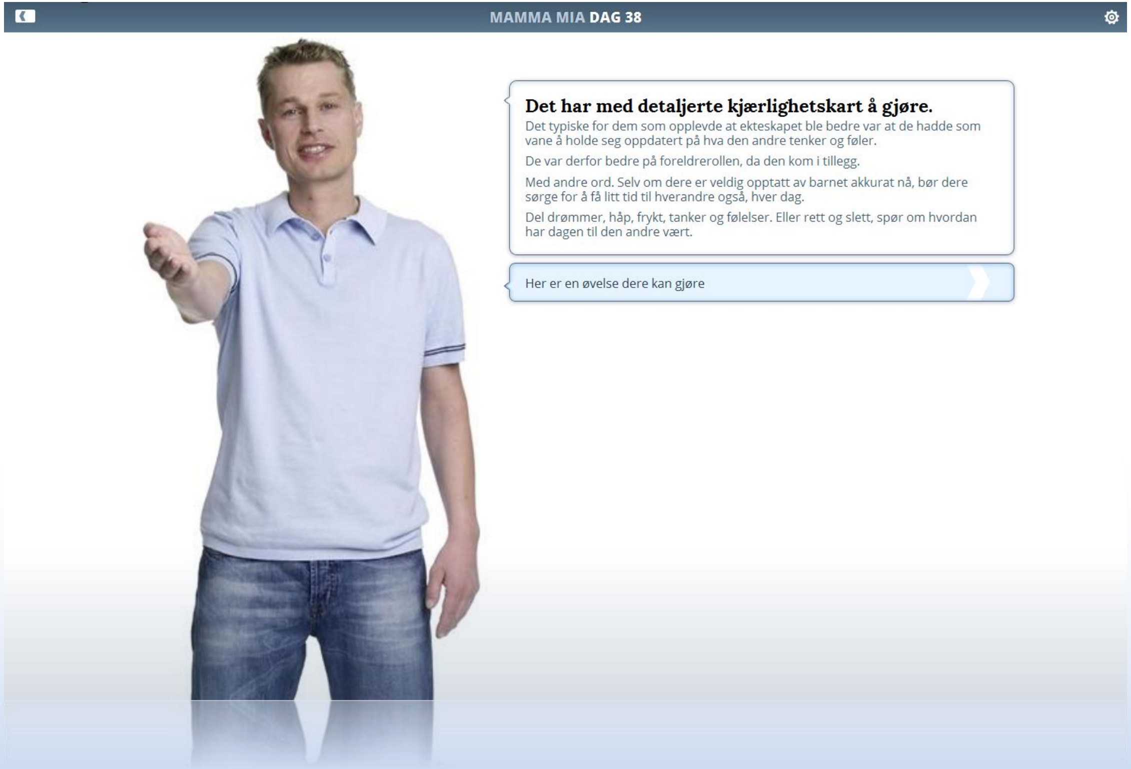

Building closeness and partner intimacy

Satisfied couples are up-to-date on each other’s inner life

Show an interest in your partner and share your thoughts and feelings

Mamma Mia

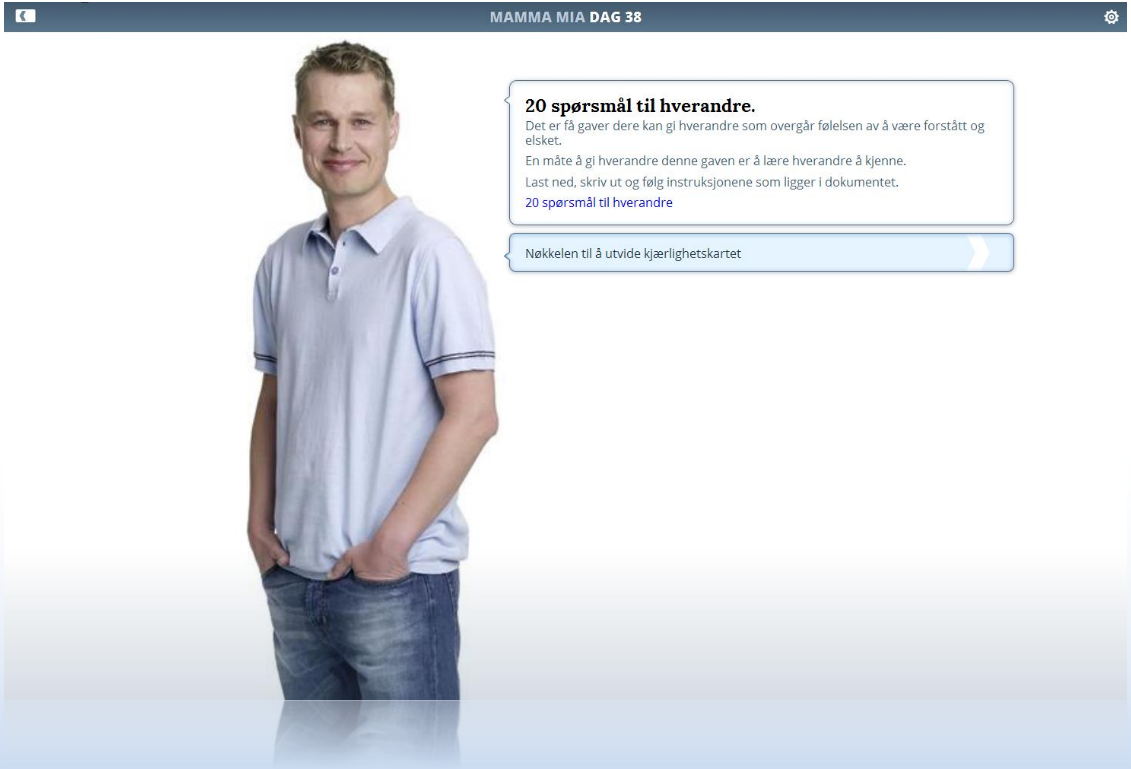

Building closeness and partner intimacy

Homework: Ask each other 20 questions to stay up-to-date

Downloadable documents to ease partner involvement

Edinburgh Postnatal Depression Scale → Metacognitive therapy → Subjective Well-Being → Couple Relationship → **Parental Sensitivity**

Getting to know your baby – Graded comfort

A way to comfort your baby when s/he is crying

Wait, watch, and wonder if the baby responds or needs more support

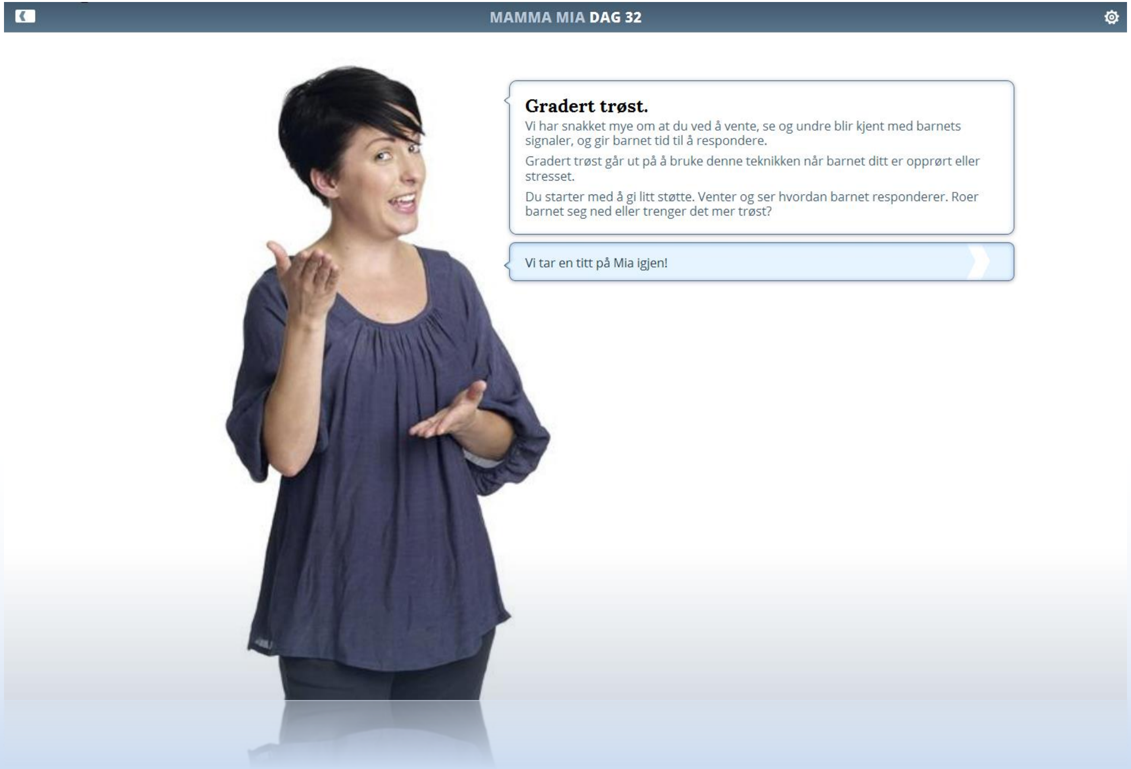

Getting to know your baby – Graded comfort

Video demonstration of the states in the sleep-wake cycle

Video demonstration of graded comforting; step-by-step

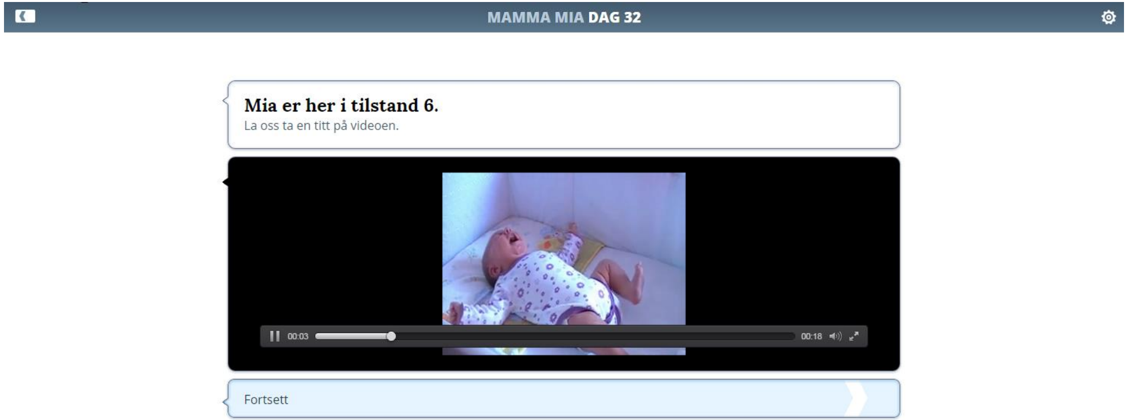

Mamma Mia

Getting to know your baby – Graded comfort

Step-by-step instructions; graded comforting explained

Reminding that each step should last 10–15 seconds

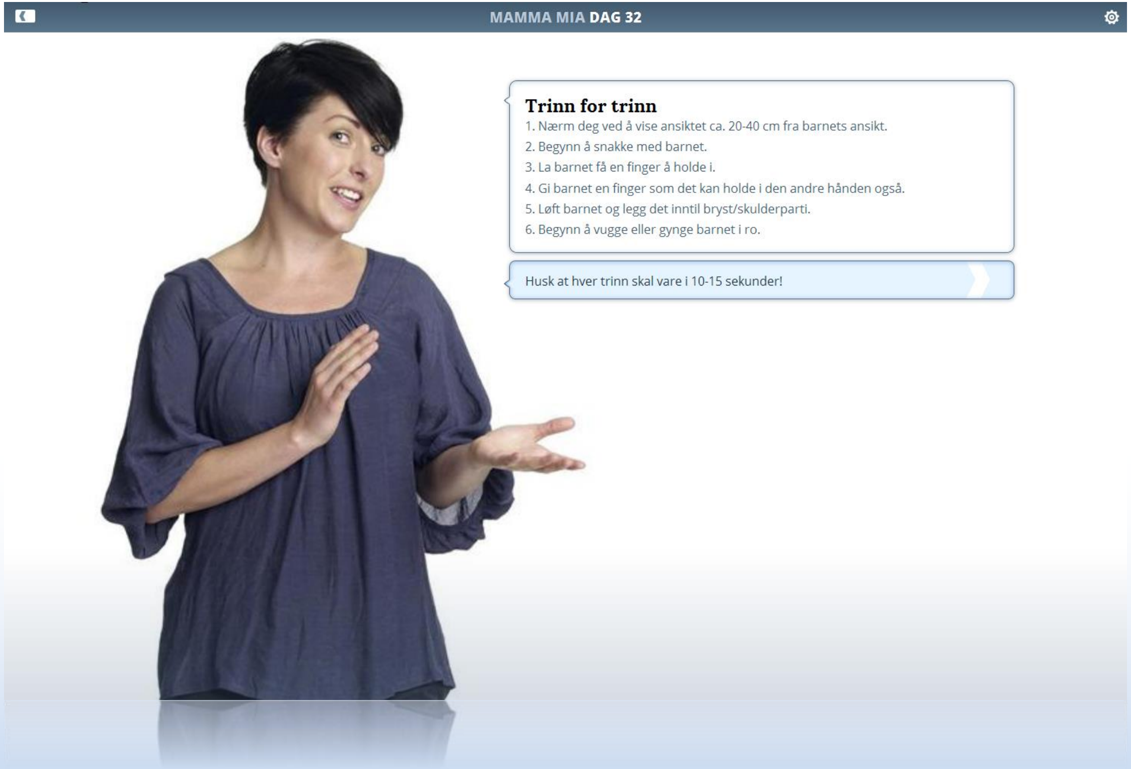

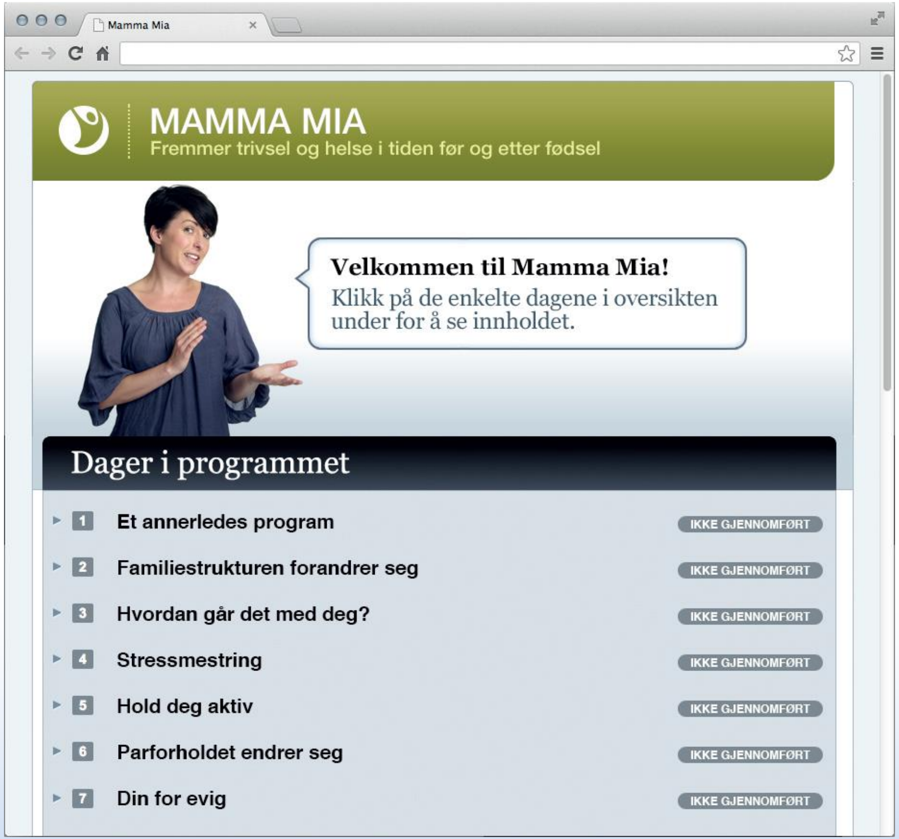

Program homepage

All program content and materials gathered in one place

24/7 access to review sessions and download materials

For a demonstration, please visit: Mamma Mia
