# Supplementary material for: An Internet-Based Intervention (Mamma Mia) for Postpartum Depression: Mapping the Development from Theory to Practice
Source: JMIR Res Protoc. 2015 Oct 12;4(4):e120. doi: 10.2196/resprot.4858 (PMC4704906; doi:10.2196/resprot.4858)
Supplement: Multimedia Appendix 2 [file resprot_v4i4e120_app2.pdf]

Multimedia Appendix 2: Transcripts*

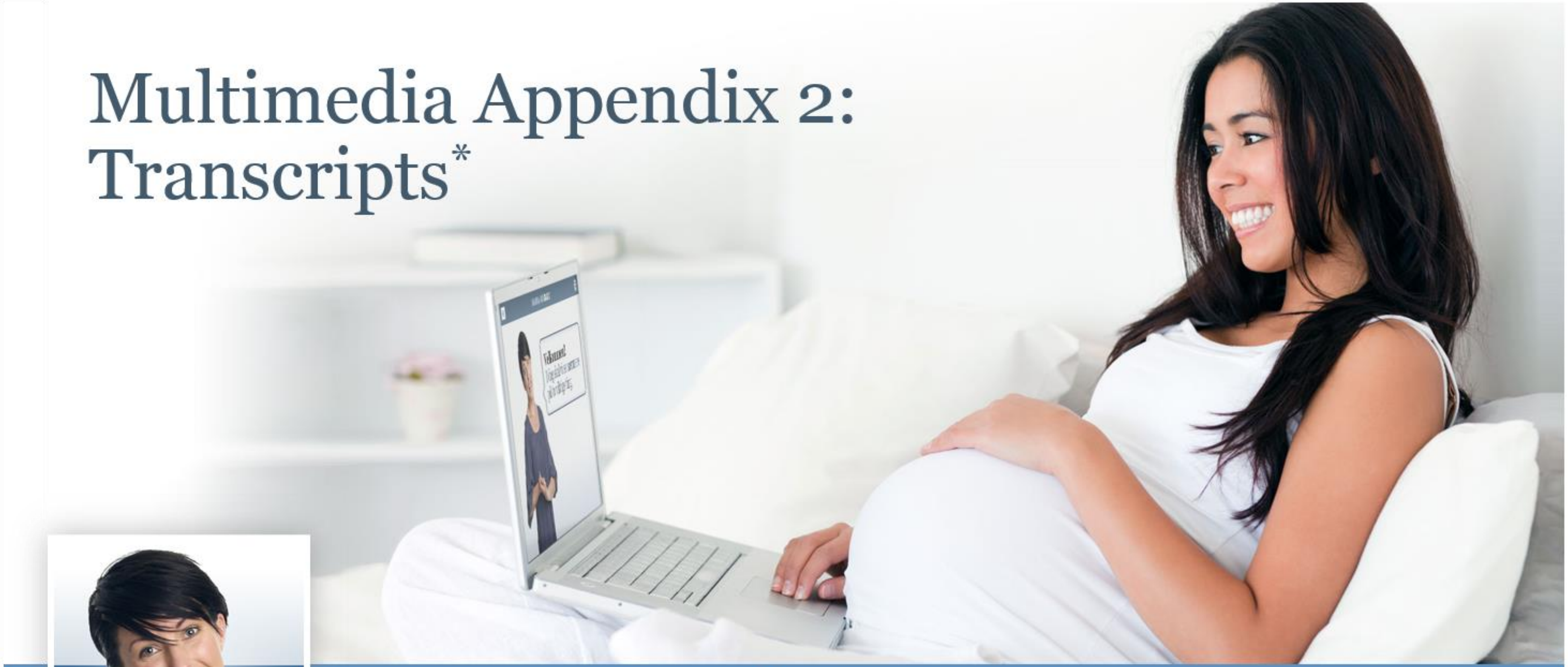

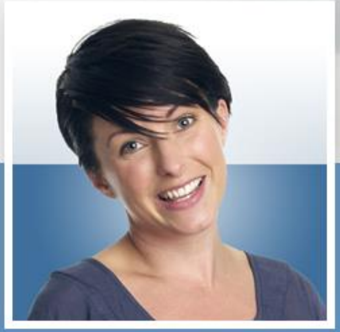

Mamma Mia

*These transcripts are excerpts from program materials to illustrate the exercises in Mamma Mia and may not fully represent the program.

Metacognitive Therapy

Attention Training Technique

These are audio-guided (i.e. mp3) instructions for performing the attention training technique and which the woman may download for personal use.

1. *Selective attention (approx. 5 minutes)*

I want you to start by focusing your gaze on a particular point in the room.
Try to keep your eyes fixed on the same point throughout the exercise.

I will ask you to focus your attention on the different sounds within and outside this room.

I will ask you to change the focus of your attention in different ways as we go along.
It does not matter if other thoughts or feelings arrive.

The goal is to practice focusing your attention on what I ask you to, regardless of what else you might become aware of, during this process.

Some sounds may be difficult to hear while other sounds may be dominant.

At first, focus on my voice [S1]. Please note the sound of my voice.
No other sound matters… Just my voice.

Try to focus all your attention on the sound of my voice. Disregard all the other sounds around you. You can hear them, but try to focus all your attention on the sound of my voice.

Focus only on my voice. Focus only on that sound. No other sound matters.

Now, you can focus your attention towards the sound of the rain [S2]. If your attention starts to wander or is caught by another sound, just turn your attention back to the rain.

Now, focus on the sound of bubbles [S3].
Beware of this particular sound… For no other sound matters.

Continue to listen to this sound and, if you are distracted, turn your attention back to this sound as soon as you can.

…

2. *Rapid attention switching (approx. 3 minutes)*

Now, that you have practiced focusing on individual sounds and spaces, I want to ask you to shift your attention quickly between sounds and spaces.

First, I ask you to focus only on the rain.

Shift your attention and focus on the sounds you can hear right behind you and nearby.

Now, turn your attention to the church bells. No other sounds matter.
Listen only to the sound of the church bells.

Shift your attention again, this time to the sound of the bubbles.

Shift again and focus on the rain.

…

3. Divided attention (approx. 2 minutes)

Finally, I want you to broaden your attention. Make it as broad and as deep as possible. Try to absorb and soak up all the sounds, all the places you have identified at the same time.

Try to simultaneously to focus on and beware of all the sounds both within and outside of this room. To the left and to the right. Count the number of sounds you can hear at the same time.

Try to hear the sounds simultaneously. Again, count the number of sounds you can hear simultaneously.

How many sounds were you able to hear at the same time?

This completes the exercise.

Subjective Well-Being

This downloadable sheet describes 18 different character strengths. The mother is asked to identify some of her strengths and plan to use these for a period in her everyday life.

Character Strengths

Instructions

Which strengths characterize you as a person? Read the list of below and write down the 3 strengths that characterize you the most and where you think:

Yes, this is really me.
Yes, this makes me enthusiastic.
Yes, this is a strength I would like to use more.
Yes, this explains how I think and act.

1. Creativity: The ability to think novel thoughts, innovate, and find solutions
- Enroll in a course in cooking, painting, photography, etc.
- Find new solutions in daily life, e.g., try a new dish for dinner several days this week

…

5. Perseverance: The ability of not giving up when something is difficult or unpleasant
- Make a to-do list and complete one task each day this week
- Focus on finishing an important task before the deadline

…

9. Social intelligence: The ability to understand people's motives and feelings
- Notice when a friend accomplishes something new and give her support and praise her for the effort
- Try to see things from the perspective of the other when you disagree with someone. Why do you think differently? What feelings and motives lie behind the disagreement?

…

Write down three of your character strengths here:

1.
2.
3.

For each of these character strengths, find something you can do every day that really allows you to utilize that particular strength even more.

You may want to use some of the examples we have given above or you may find other ways of using your character strengths to an even greater extent than before.

Couples Relationship

This is a blueprint for building closeness and intimacy with the partner. The woman is given this task as a home assignment and asked to perform the exercise at home between sessions.

Your personal love map

Instructions

Please use this blueprint to interview each other. The purpose of the exercise is to share and exchange your current thoughts and feelings.

Take turns being the interviewer and the one that is interviewed.

Do not judge or evaluate your partner, and do not provide any advice.
Your goal is simply to listen and learn as much as possible about your partner.

Questions

1. What are the most important events in your life, nowadays?

2. Of upcoming events, what are you looking most forward to and what are you dreading the most?

3. What stresses or worries you the most these days?

4. What goals, hopes, expectations or ambitions are you working towards nowadays?

5. Which persons are involved and appear important in your life now

Parental Sentivity

This is a downloadable sheet that serves, as a reminder of the characteristics of the baby's various behavioral states. The mother is encouraged to use this sheet to get to know her baby.

Sleep-Wake Cycle – States 1 & 2

Instructions

It is not always easy to observe and understand what your baby may be expressing. By learning about how infants communicate and what signals to look for, it becomes easier to understand what your baby is trying to tell you, and what your baby needs.

1. Deep sleep

Regular breathing, eyes closed, and no spontaneous movements, except for maybe small twitches, which are quickly suppressed. No eye movements under the eyelids.

2. Light sleep

Irregular breathing, random movements, and twitching eye movements under the eyelids. Give your baby time to find out whether s/he needs to sleep more or becomes ready to be picked up!

States 1 and 2 provide a fine opportunity for observing and getting to know your baby.

For a demonstration, please visit: Mamma Mia
